# Structures of LIG1 provide a mechanistic basis for understanding a lack of sugar discrimination against a ribonucleotide at the 3'-end of nick DNA

**DOI:** 10.1016/j.jbc.2024.107216

**Published:** 2024-03-24

**Authors:** Kanal Elamparithi Balu, Mitchell Gulkis, Danah Almohdar, Melike Çağlayan

**Affiliations:** Department of Biochemistry and Molecular Biology, University of Florida, Gainesville, Florida, USA

**Keywords:** DNA repair, DNA replication, DNA polymerase, DNA ligase, DNA ligation, nick sealing, ribonucleotides, sugar discrimination, genome stability

## Abstract

Human DNA ligase 1 (LIG1) is the main replicative ligase that seals Okazaki fragments during nuclear replication and finalizes DNA repair pathways by joining DNA ends of the broken strand breaks in the three steps of the ligation reaction. LIG1 can tolerate the RNA strand upstream of the nick, yet an atomic insight into the sugar discrimination mechanism by LIG1 against a ribonucleotide at the 3′-terminus of nick DNA is unknown. Here, we determined X-ray structures of LIG1/3′-RNA-DNA hybrids and captured the ligase during pre- and post-step 3 the ligation reaction. Furthermore, the overlays of 3′-rA:T and 3′-rG:C step 3 structures with step 2 structures of canonical 3′-dA:T and 3′-dG:C uncover a network of LIG1/DNA interactions through Asp570 and Arg871 side chains with 2′-OH of the ribose at nick showing a final phosphodiester bond formation and the other ligase active site residues surrounding the AMP site. Finally, we demonstrated that LIG1 can ligate the nick DNA substrates with pre-inserted 3′-ribonucleotides as efficiently as Watson-Crick base-paired ends *in vitro*. Together, our findings uncover a novel atomic insight into a lack of sugar discrimination by LIG1 and the impact of improper sugar on the nick sealing of ribonucleotides at the last step of DNA replication and repair.

The concentration of ribonucleotide triphosphates (rNTPs) is much more abundant than those of deoxyribonucleotide triphosphates (dNTPs) within a cell, and therefore, their misincorporation into genomic DNA by DNA polymerases (pols) occur at higher frequencies than mismatch nucleotide insertions, making genomic ribonucleotides the most prevalent source of cellular DNA damage (1, 2, 3, 4). In addition to locally distorting the DNA helix from a B-form to an A-form, the ribonucleotides embedded in genomic DNA can distort the DNA helix; affect the major DNA metabolisms such as replication, transcription, and repair; and can stimulate a wide range of genomic instabilities leading to an increased mutation, recombination, and chromosome alterations (5, 6, 7, 8). Ribonucleotide Excision Repair (RER), as a primary mechanism for ribonucleotide repair, is initiated by RNase H2-mediated incision of the DNA backbone at the 5′-end of the ribonucleotide, followed by strand displacement synthesis by pol δ, then flap removal by Flap Endonuclease 1 (FEN1) and/or Dna2, and finally nick sealing by DNA ligase 1 (9, 10, 11, 12). In the absence of RNase H2 activity, it has been reported that the mutation rate is increased accompanied by novel mutational events such as slippage in simple repeat sequences (13). RNase H2 mutant that cannot cleave single ribonucleotides in DNA results in persistent accumulation of ribonucleotides in genomic DNA (14). Furthermore, the mutations that impair RNase H2 function can cause the autoimmune disease Aicardi-Goutieres syndrome (AGS) and are associated with systemic lupus erythematosus (15).

In human cells, the main source of ribonucleotide contamination in DNA is their incorporation by DNA pols, which can occur during almost all cellular DNA transactions including nuclear replication and DNA repair pathways (1). DNA pols select incoming nucleotides with both the correct base and sugar moieties, preferring deoxyribose rather than ribose for incorporation during DNA synthesis. Discrimination against ribonucleotide incorporation by replicative and repair DNA pols is essential for the maintenance of genomic integrity (16). It has been extensively reported in the structure/function studies that most DNA pols exhibit a “steric gate” (*i.e*., B-, and Y- family pols) or a protein backbone segment in minor groove nucleotide-binding pocket (*i.e.*, X-family pols) which governs ribonucleotide exclusion (17, 18, 19, 20, 21, 22, 23, 24, 25, 26, 27, 28, 29, 30).

DNA ligases are fundamental enzymes to maintain the structural integrity of the human genome and seal DNA strand breaks that can occur naturally as intermediates in a wide range of DNA transactions including DNA repair, replication, and recombination (31, 32, 33). DNA ligation is the last step of almost all DNA repair pathways after nucleotide incorporation by DNA pols (34). Therefore, the fidelity of DNA synthesis that relies on incorporation of correct nucleotide by DNA polymerase is important to create a nick DNA substrate with canonical 3′-hydroxyl (OH) and 5′-phosphate (PO_4_) termini to be sealed subsequently by DNA ligase to finalize the repair pathway (35). ATP-dependent human DNA ligases, DNA ligase I, IIIα, and IV, share a conserved core architecture consisting of the oligonucleotide binding domain (OBD) and the adenylation (AdD) domains, and catalyze the conserved ligation reaction involving three consecutive chemical steps (36, 37, 38, 39): the formation of the DNA ligase-adenylate intermediate (LIG-AMP) in step 1, subsequent transfer of AMP moiety to the 5′-PO_4_ end of the nick (DNA-AMP) in step 2, and final phosphodiester bond formation coupled to AMP release in step 3.

Of the three human DNA ligases, DNA ligase 1 (LIG1) is the main replicative ligase playing a critical role in the maturation of Okazaki fragments with >50 million ligation events during DNA replication, acts in a fusion of sister chromatids by targeting double-stranded DNA breaks and is responsible for the majority of DNA ligase activity in proliferating cells (40, 41, 42, 43, 44, 45, 46). The first structure of LIG1 determined by X-ray crystallography revealed the N-terminal α-helical extension, which is recognized as a DNA-binding domain (DBD) that stimulates the relatively weak nick-sealing activity of the catalytic core (47). This first structure also demonstrated that the AdD and OBD domains interact to form a continuous protein surface that engages the minor groove of DNA and this interaction with DNA alters the substrate conformation resulting in the adoption of an RNA-like A form helix, partially unwinding the DNA duplex and positions the DNA ends at the active site for end joining (47). Furthermore, the catalytic region adopts extended and asymmetric conformation in the absence of DNA and a large conformational change occurs between AdD and OBD domains that interact with DBD to form a ligase protein clamp-like architecture that encircles a nick DNA (47).

Recently solved LIG1 structures demonstrated that the ligase employs Mg^2+^-reinforced nick DNA-binding mode to ensure high-fidelity ligation, and this accuracy is also a critical determinant of faithful replication of the nuclear genome (48, 49, 50). Furthermore, our previously solved structures revealed that the LIG1 active site discriminates against mismatches incorporated by DNA polymerase, and the ligase active site engages with mutagenic base substitution errors distinctly depending on the architecture of the 3′-terminus:template base pair at the nick (51). For example, LIG1 can accommodate G:T mismatch in a similar conformation with canonical A:T at step 2 when AMP is transferred to 5′-PO_4_ end of nick DNA, while the ligase stays adenylated at its active site lysine residue (K568) during initial step 1 of the ligation reaction (51). In the present study, we aimed to elucidate the mechanism by which human LIG1 discriminates against “wrong” sugar at the 3′-end of nick DNA at atomic resolution. This mimics the lesion with a single 3′-ribonucleotide that could be formed when repair and replication DNA polymerases incorporate rNTP into a gap repair intermediate during the DNA synthesis step.

It has been previously shown *in vitro* that LIG1 displays discrimination against the DNA substrate when the 5′-phosphorylated strand is completely RNA, while the ligase can ligate the RNA strand that is located at upstream of the nick (47). Furthermore, the ligation efficiency for the ribonucleotides at positions near the ends of nick DNA has been also reported for DNA ligases from *Chlorella* virus*, Thermus thermophilus*, and *Saccharomyces cerevisiae* (52, 53, 54, 55). Despite these studies for human and other ligases, the mechanism of sugar discrimination by human DNA ligase at atomic resolution is entirely missing.

In the present study, we investigated the features of the 3′-RNA-DNA substrate and LIG1 interaction at both biochemical and structural levels. We solved the LIG1 structures in complex with nick DNA containing a ribonucleotide at the 3′-terminus, 3′-rA:T and 3′-rG:C, showed that the ligase active site engages with these DNA-RNA heteroduplexes during pre- and post-step 3 of the ligation reaction where the product of DNA-AMP intermediate is formed and subsequent phosphodiester bond formation between 3′-OH and 5′-PO_4_ ends of nick is coupled to AMP release (Scheme S1). These structures, for the first time at atomic resolution, uncover a lack of proficient sugar discrimination by LIG1. We also solved the structure of the LIG1/nick complex with canonical 3′-dG:C end at step 2 of the ligation reaction. The overlays of LIG1 structures captured at all steps demonstrated the active site interactions, particularly through Asp(D)570 and Arg(R)871 residues, with surrounding water molecules and the 2′-OH of the ribose as well as a network of other ligase side chains surrounding the adenine group of AMP, which could be the reason for a lack of discrimination against “wrong” sugar at the 3′-end of nick by LIG1. Finally, we showed efficient ligation of nick DNA substrates containing 3′-ribonucleotides 3′-rA:T, 3′-rG:C, and 3′-rC:G, which was found to be similar to the ligation of canonical 3′-dA:T, 3′-dG:C, and 3′-dC:G ends *in vitro*. LIG3α also exhibits this feature of efficient end-joining ability for nick DNA substrates with 3′-ribonucleotides. Overall, our findings provide a novel and atomic insight into the characterization of ribonucleotide selectivity at the 3′-terminus of nick DNA by LIG1, demonstrating its inability to surveil an incorrect sugar on the downstream events of DNA replication and repair.

## Results

### Structures of LIG1/nick DNA complexes with a ribonucleotide during the last step of the ligation reaction

**Figure 1:**
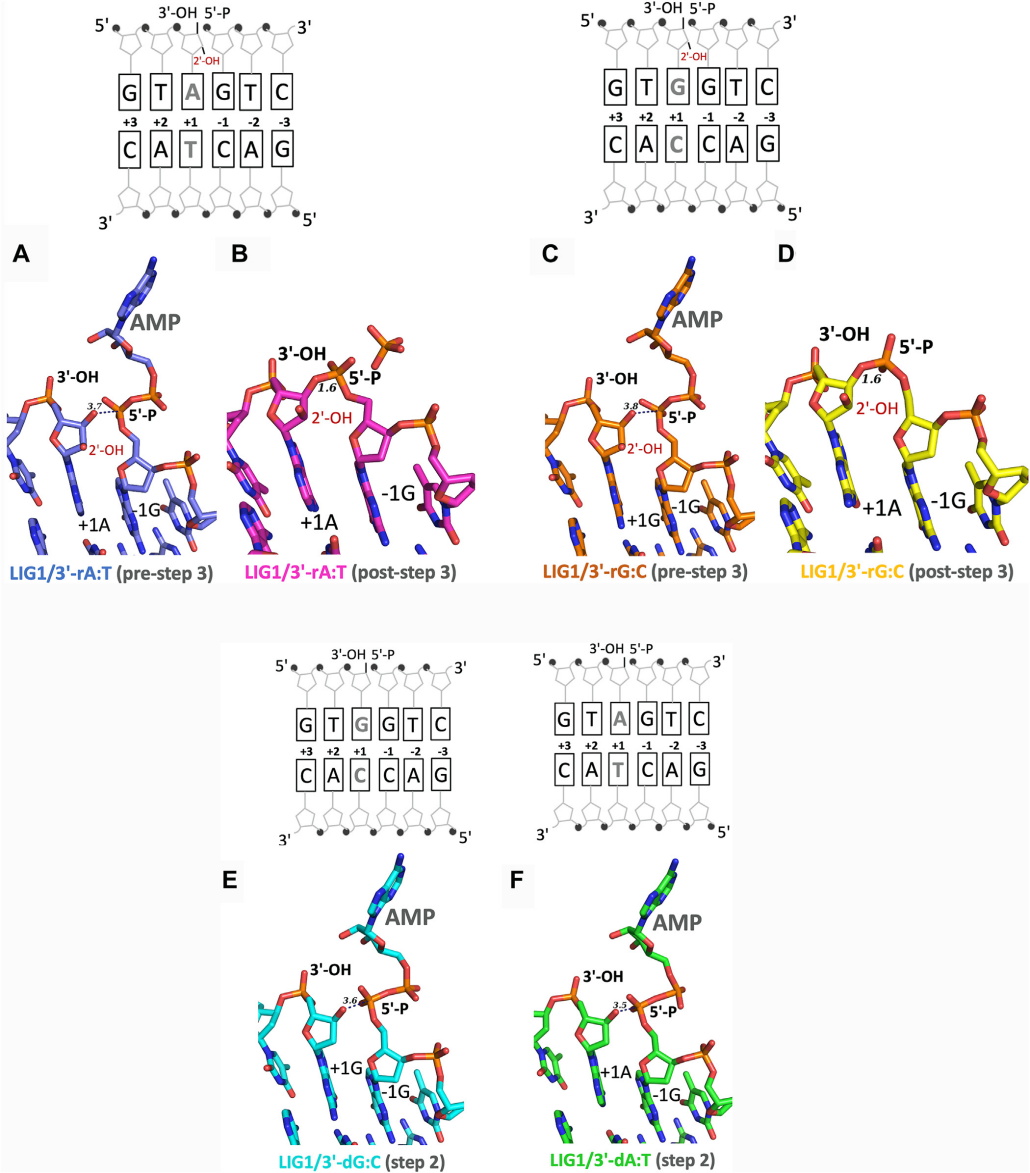
**Structures of LIG1 bound to nick DNA complexes.***A*−*D*, structures of LIG1 in complex with nick DNA containing 3′-ribonucleotides at pre- and post-step 3 of the ligation reaction for rA:T (*A* and *B*) and 3′-rG:C (*C* and *D*). *E* and *F*, structures of LIG1 in complex with nick DNA containing 3′-dG:C (*E*) and 3′-dA:T (*F*). A schematic view of the DNA substrate used in the LIG1 crystallization shows the sequence of 3′- and 5′-ends at the nick site. LIG1/3′-dA:T structure is previously solved (PDB: 7SUM).

For LIG1 crystallization, we used the EE/AA mutant that harbors E346A and E592A mutations, resulting in the ablation of the high-fidelity site (referred to as Mg^HiFi^ site), which has been utilized in previous LIG1 structures with non-canonical substrates (48, 49, 50, 51). We determined X-ray structures of LIG1 in complex with the nick DNA containing 3′-ribonucleotides (3′-rA:T and 3′-rG:C) as well as 3′-dG:C (Table 1 and Fig. 1). LIG1/DNA-RNA heteroduplexes were captured at the pre- and post-step 3 of the ligation reaction, while we observed LIG1/nick DNA complex containing canonical 3′-dG:C at step 2 (Scheme S1).

**Table 1:** X-ray data collection and refinement statistics of LIG1 structures

PDB entry ID	LIG1^EE/AA^ 3'-rA:T (post-step 3) 8VDT	LIG1^EE/AA^ 3'-rG:C (post-step 3) 8VDS	LIG1^EE/AA^ 3'-dG:C (step 2) 8VDN	LIG1^EE/AA^ 3'-rA:T (pre-step 3) 8VZM	LIG1^EE/AA^ 3'-rG:C (pre-step 3) 8VZL
Data collection					
Space group	P2_1_2_1_2_1_	P2_1_2_1_2_1_	P2_1_2_1_2_1_	P2_1_2_1_2_1_	P2_1_2_1_2_1_
Cell dimensions					
*a*, *b*, *c* (Å)	64.1, 115.3, 125.0	64.7, 115.9, 126.2	64.9, 115.6, 126.1	64.8, 115.9, 123.0	65.0, 115.2, 123.9
α, β, γ (°)	90	90	90	90	90
Resolution (Å)	24.83–2.80 (2.852.80)	20–2.80 (2.85–2.80)	40–2.39 (2.44–2.39)	25–2.5 (2.54–2.51)	25–2.41 (2.34–2.41)
*Rpim*	0.042 (0.378)	0.033 (0.310)	0.031 (0.510)	0.040 (0.394)	0.041 (0.447)
*I*/σ (*I*)	14.9 (1.44)	24.5 (1.37)	17.1 (1.6)	27.3 (1.6)	28.1 (1.5)
*CC*_*1/2*_	0.99 (0.803)	0.98 (0.87)	0.994 (0.673)	0.993 (0.693)	0.993 (0.646)
*CC*	0.99 (0.944)	0.99 (0.96)	0.998 (0.897)	0.998 (0.905)	0.993 (0.886)
Completeness (%)	98.5 (99.7)	99.8 (100.0)	99.4 (100.0)	99.6 (100.0)	98.7 (94.8)
Redundancy	6.1 (6.6)	12.7 (13.2)	5.9 (6.6)	6.1 (6.4)	6.5 (5.7)
Wilson B-factor	77.85	74.06	66.84	60.00	52.7
Refinement					
Resolution (Å)	24.83–2.78	19.90–2.79	39.52–2.39	25–2.51	25–2.41
No. reflections	23,397	23,884	37,843	32,094	35,822
*R*_work_/*R*_free_	20.3/24.9	20.1/25.2	20.7/24.1	17.9/23.3	17.9/22.0
Non-H atoms	5296	5503	5505	5814	5916
Protein	4553	4756	4712	4910	4926
DNA/RNA	733	733	732	733	733
AMP	-	-	23	23	23
H_2_O	25	14	38	148	234
Average B- factor (Å2)	91.92	90.06	85.23	74.54	65.90
Protein	96.11	94.02	89.24	78.10	68.92
DNA	66.46	64.75	61.01	56.10	47.88
Ligand	113.38	-	74.48	65.02	50.72
H_2_O	74.21	70.37	60.73	65.48	60.22
R.M.S.D					
Bond lengths (Å)	0.002	0.003	0.002	0.002	0.002
Bond angles (°)	1.221	0.552	0.554	0.542	0.539

In the pre-step 3 structures of 3′-rA:T (Fig. 1*A*) and 3′-rG:C (Fig. 1*C*), the product of DNA-AMP complex is formed when the AMP moiety is transferred to the 5′-end of the nick DNA. However, in the post-step 3 structures of LIG1/3′-rA:T (Fig. 1*B*) and 3′-rG:C (Fig. 1*D*), the 3′-OH terminus attacks the 5′-PO_4_ terminus downstream of the nick DNA and a phosphodiester bond is formed. In addition, LIG1/3′-dG:C structure shows the formation of DNA-AMP intermediate referring to step 2 of the ligation reaction (Fig. 1*E*), which is similar to our previously solved LIG1/3′-dA:T structure (PDB: 7SUM) (Fig. 1*F*) and LIG1 structures for canonical 3′-dG:C and damaged 3′-8oxodG:A as reported by others (Table S1) (48, 49, 50).

When we compared the density for AMP in the pre- *versus* post-step 3 structures of 3′-rA:T and 3′-rG:C (Fig. S1), the omit Fo-Fc maps at 3σ of the AMP moiety shows good density for almost all the atoms in the AMP in the pre-step 3 structures while only sparse density is observed in the post-step 3 structures. This suggests that the AMP is released which occurs after the formation of a phosphodiester bond between DNA ends. The omitted Fo-Fc map revealed continuous density between the 3′-OH and 5′-PO_4_ ends of the nick. However, the density between the nick termini is greater in the post-step 3 structures, consistent with covalent bond formation, while this density is weak in the pre-step 3 structures, consistent with hydrogen bond formation or density overlap due to our moderate resolution.

The overlay of the pre- and post-step 3 structures of 3′-rA:T and 3′-rG:C (Fig. S2, *A* and *B*) demonstrated the difference in the position of AMP as well as in the distance between 3′-OH and 5′-PO_4_ ends of nick, which were measured to be 3.7 *versus* 1.6 Å (3′-rA:T) and 3.8 *versus* 1.6 Å (3′-rG:C) in the pre- *versus* post-step 3 structures. From the superposition of step 2 and pre-step 3 structures of 3′-deoxyribonucleotides *versus* 3′-ribonucleotides (Fig. S2, *C* and *D*), the distance between 3′-OH and 5′-PO_4_ of nick were measured to be 3.7Å (3′-rA:T) and 3.8Å (3′-rG:C) *versus* 3.5Å (3′-dAT) and 3.6Å (3′-dG:C), revealing that the difference in the distance between DNA ends containing canonical base-pair and ribonucleotide is constant (0.2Å). Furthermore, the overlays of the step 2 structures of 3′-dA:T and 3′-dG:C *versus* the post-step 2 structures of 3′-rA:T and 3′-rG:C (Fig. S2, *E* and *F*), showed bond angle changes at the 5′-PO_4_-AMP linkage, which were calculated to be 148.3° and 150.9° for the structures of 3′-deoxyribonucleotides and 129.9° and 127.6° for the structures of 3′-ribonucleotides.

Finally, we observed the Watson-Crick base pair for all LIG1/nick DNA structures containing 3′-ribonucleotides and sugar pucker analyses demonstrate that the ribose adopts C3′-endo conformation at the 3′-end of nick (Fig. S3 and S4). The root-mean-square deviation (RMSD) of the LIG1 structures was no higher than 0.97 Å for all main chain atoms (Table S2). The superimposition of all six LIG1 structures with and without 3′-ribonucleotide show a global conformation of the catalytic core consisting of AdD, DBD, and OBD domains that encircle nick DNA (Fig. S5).

### LIG1 fails to discriminate against a ribonucleotide at the 3′-end of nick DNA

**Figure 2:**
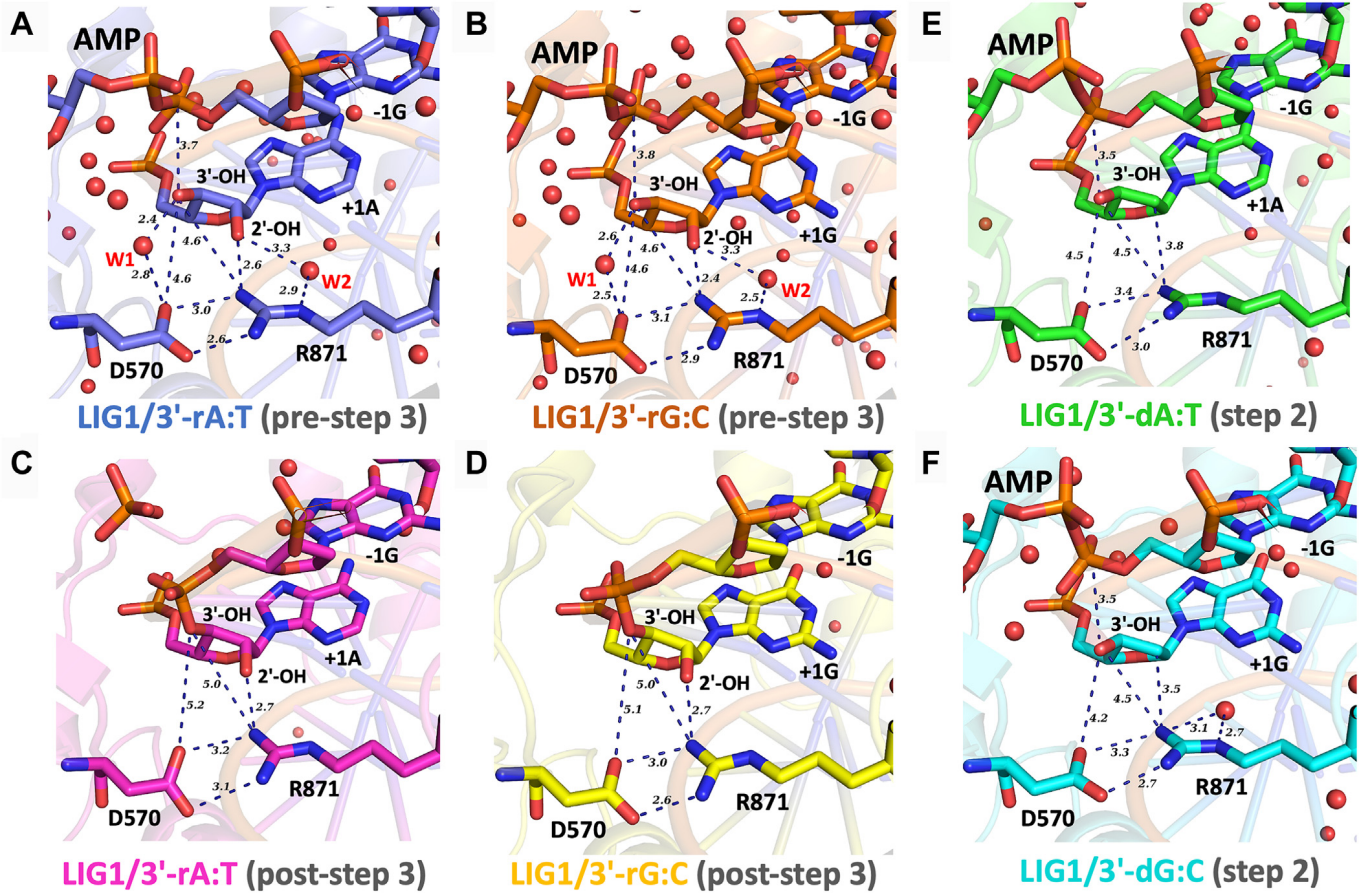
**LIG1 structures show the interaction network of the critical active site residues.** The interactions are shown between LIG1 active site amino acid residues D570 and R871, the 2′-hydroxyl group (2′-OH), and water molecules (W1 and W2) at the nick site in the structures of pre- and post-step 3 of 3′-rA:T and 3′-rG:C (*A−D*) and the step 2 of 3′-dA:T and 3′-dG:C (*E* and *F*).

We then analyzed LIG1active site around the 3′-nick site containing the 2′-hydroxyl group (2′-OH) of the ribose in the pre- and post-step 3 structures of 3′-ribonucleotides (3′-rA:T and 3′-rG:C), and observed the central differences in the interaction network of the active site residues Asp(D)570 and Arg(R)871 (Fig. 2). In the pre-step 3 structures of 3′-rA:T and 3′-rG:C (Fig. 2, *A* and *B*), R871 forms a hydrogen bond with the 2′-OH of the ribose and D570 interacts with the 3′-OH through water molecules. Yet, this interaction does not exist in the post-step 3 structures of 3′-rA:T and 3′-rG:C (Fig. 2, *C* and *D*). In step 2 structures of canonical ends, 3′-dA:T and 3′-dG:C, there is no hydrogen bond formation we observed with both side chains (Fig. 2, *E* and *F*).

**Figure 3:**
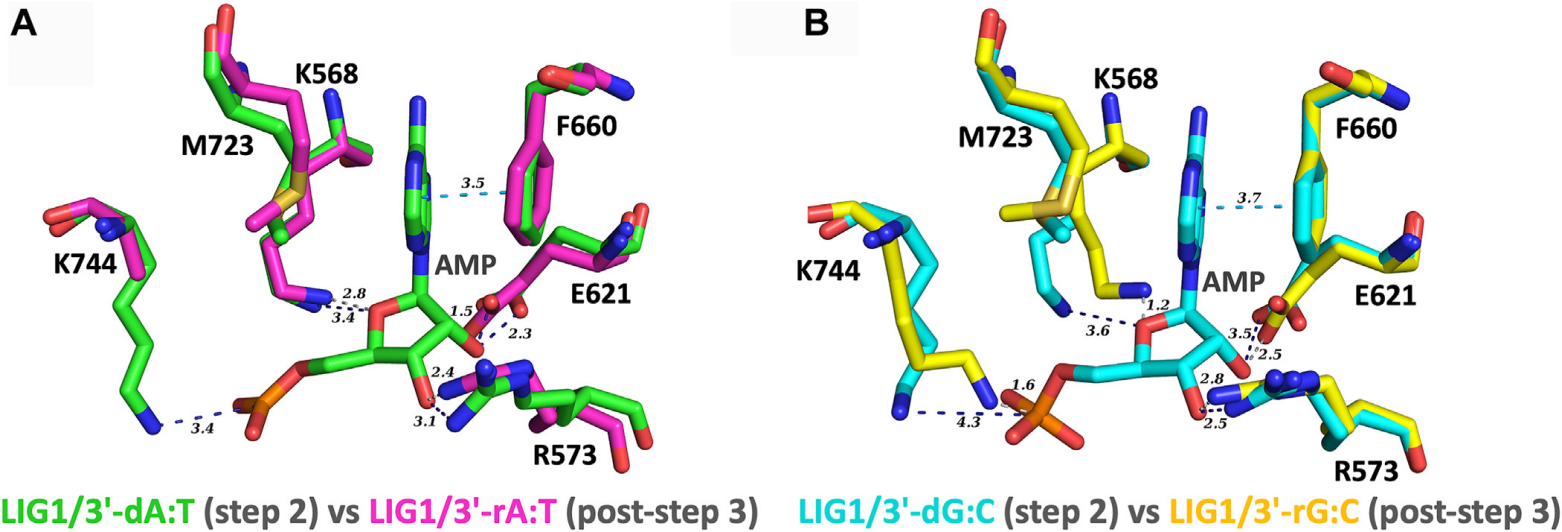
**Structural overlays show the differences in the conformational changes at the active site residues surrounding AMP.** The superimposition of LIG1 structures at step 2 and post-step 3, 3′-dA:T *versus* 3′-rA:T (*A*) and 3′-dG:C *versus* 3′-rG:C (*B*), reveal the release of AMP due to the flexible amino acids around AMP occupy the position of AMP site in the post-step 3 structures.

Furthermore, the structural overlays of the step 2 structures containing canonical ends and the post-step 3 structures containing ribonucleotides highlighted the differences in the positions of the ligase side chains surrounding the AMP site (Fig. 3). In the step 2 structures of LIG1/3′-dA:T and 3′-dG:C, the adenine group of AMP is sandwiched between Phe(F)660 and Met(M)723 amino acid residues *via* pi-pi interaction with F660. We also observed that 2′-OH of the ribose on AMP interacts with the side chains of Glu(E)621 and Arg(R)573 through hydrogen bonds. In the post-step 3 structures of 3′-rA:T and 3′-rG:C, E621 residue shifted closer by ∼1 Å toward the 5′-PO_4_ (+1G) of the nick that is sealed through a phosphodiester bond formation. Also, the side chain rotation was observed at E621 by 25.6° between the step 2 structure of LIG1/3′-dA:T and the post-step 3 structure of LIG1/3′-rA:T. Similarly, we observed the side chain rotation by 30.1° between the step 2 structure of LIG1/3′-dG:C and post-step 3 structure of LIG1/3′-rG:C. Furthermore, in the post-step 3 structure of LIG/3′-rA:T, R573 moves closer to AMP by 0.8 Å and rotates by 33.7˚. The sidechain rotation by 24.5° was observed at R573 in the post-step 3 structure of LIG1/3′-rG:C as well. We could not locate the side chain K744 in the electron density for the post-step 3 structure of LIG1/3′-rA:T and witness the side chain movement of ∼ 3 Å toward the phosphate group of AMP in the post-step 3 structure of LIG1/3′-rG:C. The active site amino acid residue K568 of LIG1 to be adenylated during initial step 1 of the ligation reaction has significant side chain movement in both post-step 3 structures compared to the step 2 structures with canonical ends. The distance between the amine group of K568 and the ether group of ribose sugar in AMP was calculated to be 2.8 and 1.2 Å in the post-step 3 structures of 3′-rA:T and 3′-rG:C, respectively. The distance between the AMP ether group and K568 is measured to be 3.6 Å in the step 2 structure of 3′-dG:C.

Overall, these findings showed a cooperative network of LIG1 active site positively charged R871 interactions with 2′-OH of the ribose and the water bridge between D570 and 3′-OH forms a phosphate diester bond by condensation reaction. This could provide atomic insight into the understanding of a lack of sugar discrimination against a single ribonucleotide on the 3′-hydroxyl side of the nick by LIG1 (Scheme S2).

### Ligation efficiency of nick DNA containing a ribonucleotide at the 3′-end

**Figure 4:**
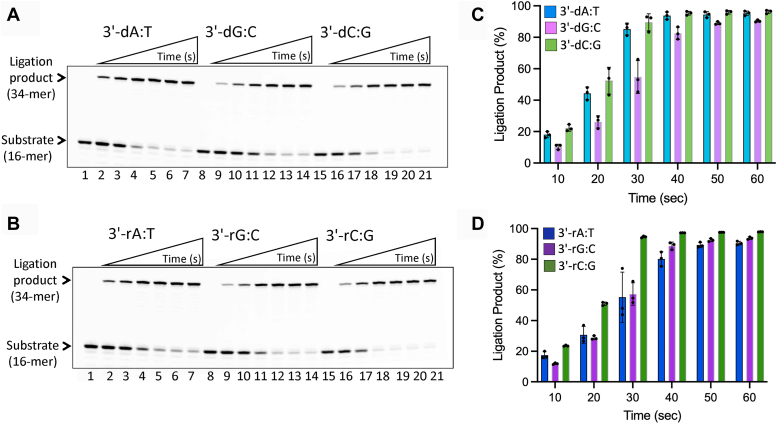
**Ligation of the nick DNA substrates with preinserted ribonucleotides at the 3′-end by LIG1.***A*, lanes 1, 8, and 15 are the negative enzyme controls of the nick DNA substrates, and lanes 2 to 7, 9 to 14, and 16 to 21 are the ligation products in the presence of 3′-dA:T, 3′-dG:C, and 3′-dC:G, respectively, by LIG1, and correspond to time points of 10, 20, 30, 40, 50, and 60 s. *B*, lanes 1, 8 and 15 are the negative enzyme controls of the nick DNA substrates and lanes 2 to 7, 9 to 14, and 16 to 21 are the ligation products in the presence of 3′-rA:T, 3′-rG:C, and 3′-rC:G, respectively, by LIG1, and correspond to time points of 10, 20, 30, 40, 50, and 60 s. *C* and *D*, graphs show the time-dependent change in the amount of ligation products for nick DNA substrates containing 3′-deoxyribonucleotides (*C*) *versus* 3′-ribonucleotides (*D*). The data represent the average from three independent experiments ± SD.

**Figure 5:**
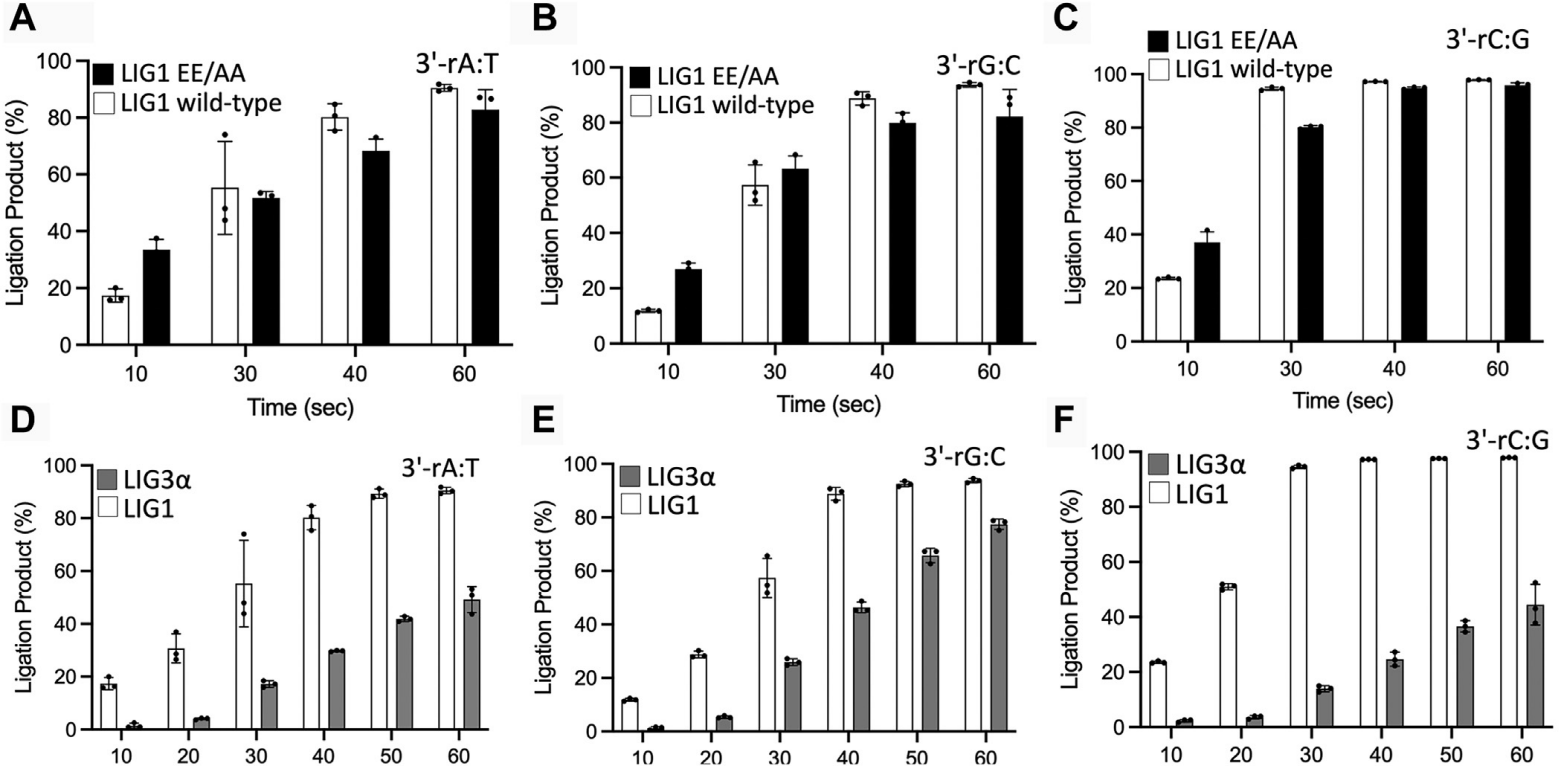
**Comparisons for the ligation efficiency of nick DNA substrates containing 3′-ribonucleotides.** Graphs show the time-dependent change in the amount of ligation products for nick DNA substrates containing 3′-deoxyribonucleotides and differences in the nick sealing efficiency between LIG1 wild-type and low-fidelity EE/AA mutant (*A−C*) and between LIG1 and LIG3α (*D−F*). The data represent the average from three independent experiments ± SD.

We then investigated the ligation efficiency of LIG1 for the nick DNA substrates containing preinserted single ribonucleotide at the 3′-end *in vitro* (Scheme S3). As expected, in the control ligation assays, we obtained efficient nick sealing of canonical ends 3′-dA:T, 3′-dG:C, and 3′-dC:G (Fig. 4*A*). Similarly, our results demonstrated very efficient ligation of the nick DNA substrates containing 3′-rA:T, 3′-rG:C, and 3′-rC:G (Fig. 4*B*). There was no major difference in the amount of ligation products between nick DNA substrates with 3′-deoxyribonucleotides *versus* 3′-ribonucleotides (Fig. 4, *C* and *D*). We then compared the ligation efficiency of the LIG1 EE/AA mutant that was used in LIG1 crystallization. Our results demonstrated similar ligation profile for the nick DNA substrates containing 3′-rA:T, 3′-rG:C, and 3′-rC:G as efficient as the nick sealing of canonical ends in the presence of the low-fidelity ligase (Fig. S6). We did not observe significant difference in the ligation efficiency between LIG1 wild-type and EE/AA suggesting that the double mutation at Mg^HiFi^ site has no impact on sugar discrimination (Fig. 5, *A*–*C*).

**Figure 6:**
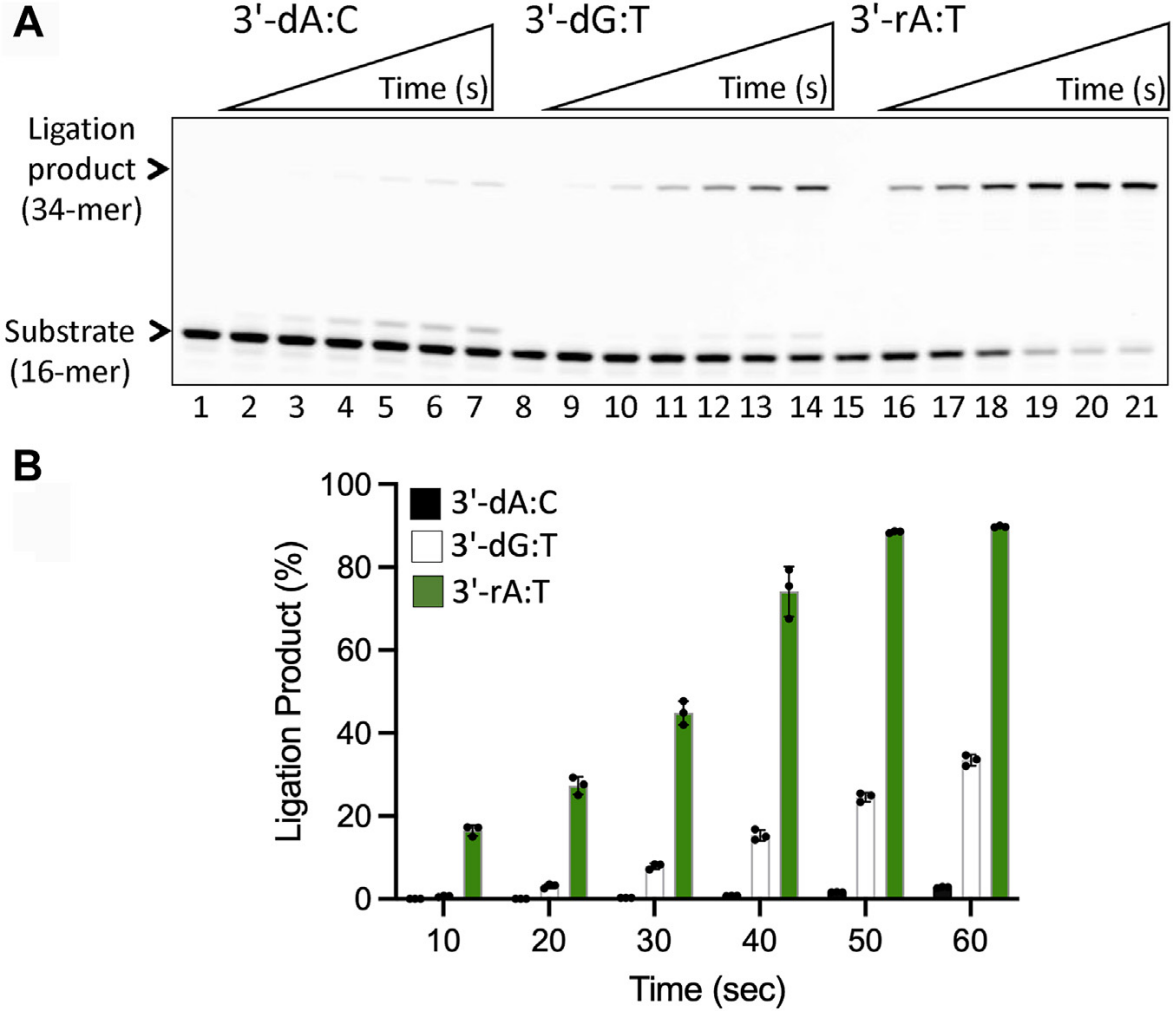
**Ligation of the nick DNA substrates with mismatch *versus* ribonucleotide at the 3′-end by LIG1.***A*, lanes 1, 8 and 15 are the negative enzyme controls of the nick DNA substrates and lanes 2 to 7, 9 to 14, and 16 to 21 are the ligation products in the presence of 3′-dA:C, 3′-dG:T, and 3′-rA:T, respectively, and correspond to time points of 10, 20, 30, 40, 50, and 60 s. *B*, graph shows time-dependent change in the amount of ligation products and the data represent the average from three independent experiments ± SD.

We previously reported that LIG1 discriminates base substitution errors introduced by repair DNA polymerase distinctly depending on the 3′-terminus/template base pairs at the nick site, and our LIG1/mismatch structures revealed that the ligase active site engages with mismatches at different ligation steps, *i.e.*, A:C and G:T at step 1 and step 2, respectively (51, 56). In the present study, we compared the ligation efficiency of nick DNA substrates with those mismatches and ribonucleotide (Fig. 6). Our results showed relatively less efficient ligation for 3′-dA:C and 3′-dG:T mismatches in comparison with the nick sealing of 3′-rA:T substrate (Fig. 6*A*). The amount of ligation products demonstrated ∼90-fold difference in nick-sealing efficiency by the effect of a ribonucleotide at the 3′-end of nick (Fig. 6*B*).

We used the low-fidelity EE/AA double-mutant of LIG1 to crystalize the protein/nick DNA complex in conditions lacking Mg^2+^. Accordingly, we confirmed the presence of double-mutation at the Mg^HiFi^ site, particularly at E346 and E592 amino acid residues in the LIG1/3′-rA:T structure (Fig. S7). Furthermore, using this crystal mutant of LIG1, we tested the impact of Mg^2+^ on the efficient ligation of ribonucleotide-containing nick DNA substrates we observed. Our results showed that LIG1 EE/AA can seal nick DNA with 3′-rA:T, 3′-rG:C, and 3′-rC:G and the yield was relatively less in comparison to the ligation products in the reaction conditions including Mg^2+^ (Fig. S8).

### Comparison of nick sealing efficiency in the presence of 3′-ribonucleotide by LIG1 *versus* LIG3α

**Figure 7:**
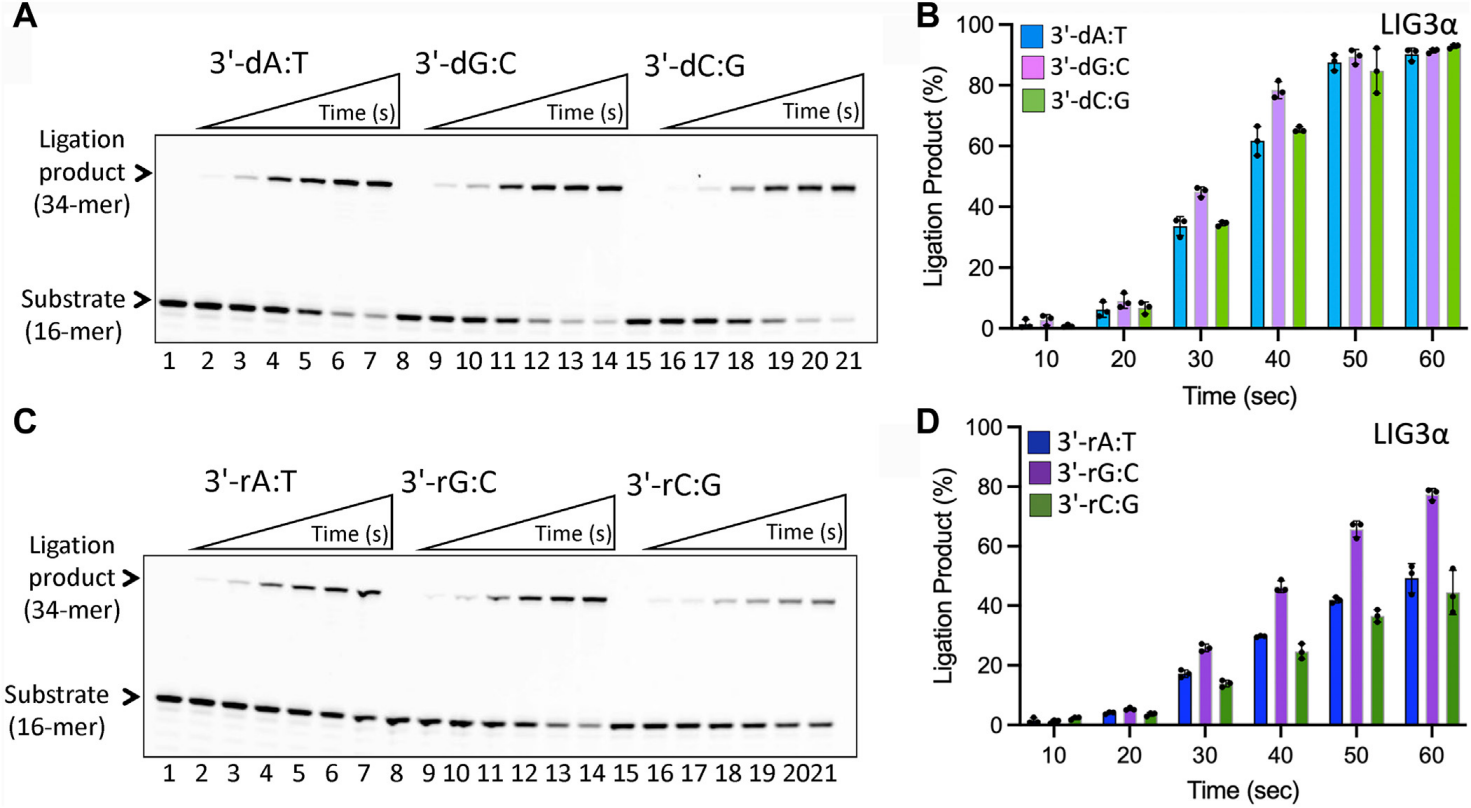
**Ligation of the nick DNA substrates with 3′-ribonucleotides by LIG3α.***A* and *C*, lanes 1, 8 and 15 are the negative enzyme controls of the nick DNA substrates. Lanes 2 to 7, 9 to 14, and 16 to 21 are the ligation products in the presence of 3′-dA:T, 3′-dG:C, 3′-dC:G, respectively (*A*), and 3′-rA:T, 3′-rG:C, 3′-rC:G, respectively (*C*), by LIG3α, and correspond to time points of 10, 20, 30, 40, 50, and 60 s. *B* and *D*, Graphs show the time-dependent change in the amount of ligation products for nick DNA substrates containing 3′-deoxyribonucleotides (*B*) *versus* 3′-ribonucleotides (*D*). The data represent the average from three independent experiments ± SD.

LIG1 and LIG3α catalyze the conserved ligation mechanism and share a high degree of structural conservation within their three-domain architecture (33). We previously reported that both ligases display distinct fidelity for nick DNA substrates containing all possible 12 non-canonical mismatches at the 3′-end (56). Finally, in the present study, we compared the ligation efficiency of LIG1 and LIG3α for nick substrates containing a single ribonucleotide (Fig. 7). Our results demonstrated that LIG3α can also ligate the nick DNA substrates 3′-rA:T, 3′-rG:C, and 3′-rC:G (Fig. 7, *C* and *D*) as efficiently as the canonical substrates 3′-dA:T, 3′-dG:C, and 3′-dC:G (Fig. 7, *A* and *B*). The comparison of the ligation efficiency with LIG1 demonstrated less efficient nick sealing of 3′-ribonucleotide-containing substrates by LIG3α (Fig. 5, *D*–*F*), suggesting that minor changes in the active site architecture of LIG1 *versus* LIG3α contribute to differences in the ribonucleotide discrimination mechanism of human ligase.

## Discussion

The contamination of DNA with ribonucleotides, which are RNA precursors, exceeds all other known types of DNA damage, which is influenced by the great excess of ribonucleotides over deoxyribonucleotides in cells (1, 2, 3). If left unrepaired, ribonucleotides confound the structural and chemical integrity of duplex DNA, increase its susceptibility to endogenous or exogenous damage, and lead to several types of genome instability such as replication blockage, mutagenesis, aberrant recombination, protein-DNA crosslinks, double-strand breaks, and chromosome alterations (5). The most common source of ribonucleotides embedded into genomic DNA is through their incorporation by almost all eukaryotic DNA polymerases during replication and repair in both nucleus and mitochondria (8). Although they can accurately discriminate the correct substrate dNTPs against rNTPs, the rate of ribonucleotide incorporation by DNA polymerases is much higher than those of mismatches as shown *in vivo* and *in vitro* for the genomes of yeast and mouse embryonic fibroblast cells (16). Extensive structural studies have provided important mechanistic insights into how these enzymes discriminate incoming nucleotides according to both the correct base and sugar moiety and select the nucleotide containing the correct sugar for incorporation into DNA (18, 19, 20, 21, 22, 23, 24, 25, 26, 27, 28, 29, 30).

DNA strand breaks occur naturally during nuclear replication machinery and a complex network of DNA repair pathways. DNA ligases ligate breaks in the backbone structure of DNA double-helix in almost every aspect of DNA metabolism in living cells and nicks, as deleterious DNA lesions, need to be faithfully sealed by DNA ligases after nucleotide incorporations by DNA polymerases at the last step of DNA repair pathways (32). Although molecular mechanisms underlying ribonucleotide-induced genome instability have been of great interest and ribonucleotide selectivity has been largely studied at the atomic level from across all families of eukaryotic DNA pols, it’s unknown how human DNA ligases achieve sugar specificity over undamaged nicks with proper Watson-Crick base pairing at atomic resolution. It has been known that DNA ligases have evolved a tolerance for ribonucleotides at the 3′-end while heavily discriminating against a ribonucleotide at the 5′-end and the first X-ray crystal structure of LIG1 revealed that the ligase enforces an A-form-like helical structure upstream of the nick (47). Although our previously solved LIG1 structures and others demonstrated the mechanism by which LIG1 discriminates against nicks containing damaged or mismatched bases at the 3′-terminus (48, 49, 50, 51), structural insight into the mechanism of sugar discrimination at the 3′-end of nick DNA by LIG1 is entirely missing.

Our LIG1 structures of RNA-DNA substrate complexes mimic mutagenic repair intermediate that could be formed due to the repair or replication of DNA polymerase-mediated ribonucleotide incorporation before the final nick-sealing step by human DNA ligases during almost all DNA repair mechanisms and DNA replication. Our structures represent the first structures of human DNA ligase captured at that final stage (pre- and post-step 3), which further contributes to the atomic-level understanding of the nick-sealing mechanism by LIG1 (Table S1).

LIG1 structures captured in the crystal conditions without Mg^2+^ show 3′-endo conformation of sugar pucker that ribonucleotides naturally adopt. In B-form DNA, the sugar moieties usually adopt the 2′-endo sugar pucker conformation, while in A-form DNA or RNA duplexes the sugar moieties usually adopt the 3′-endo sugar pucker conformation (57). The ligase active site forces the DNA upstream of the nick to adopt an A-form conformation which causes the sugar at the 3′-end to assume the 3′-endo conformation. Mg^2+^ can activate the 3′-OH for nucleophilic attack on the 5′-P and the 3′-end of nick DNA can also stabilize the 3′-endo conformation. Furthermore, in the presence of a 3′-ribonucleotide, a phosphate diester bond could form by the condensation reaction between 3′- and 5′-ends of the nick. We observed in the post-step 3 structures of 3′-ribonucleotides that the water bridge between D570 and 3′-OH as well as the interaction between 2′-OH and the sidechain of R871 provide possible chemical conformation for the condensation reaction. The water molecule between 3′-OH and D570 may form carbocation at 3′-end, while the interaction of R871 creates electron localization at 2′-C leading to form a pi bond between 2′-and 3' carbons, which in turn results in the nucleophilic attack. Furthermore, from the structural overlays of LIG1 structures with 3′-deoxyribonucleotides (step 2) and 3′-ribonucleotides (post-step 3), we observed significant arrangements and reordering of the ligase active sites that occur after phosphodiester bond formation. There was a shift in K568, R573, K744, and E621 in the post-step 3 structures of 3′-rA:T and 3′-rG:C, which would clash all over with AMP. Because of this, we suggest that E621 may reposition after phosphodiester bond formation to facilitate AMP release. In addition, the overlays of pre- and post-step 3 structures of LIG1/3′-rA:T and 3′-rG:C uncover that the side chains D570 and R871 govern critical interactions with water molecule and 2′-OH of the ribose at the 3′-end of nick site. Overall, this network of interactions at the ligase active site and conformational differences at the side chains around the AMP could be the reason for a lack of sugar discrimination and efficient ligation of nick DNA with a single ribonucleotide at the 3′-end by LIG1 (Scheme S2). Further side-directed mutagenesis studies at these active site residues are required to better understand their contribution to nick sealing mechanism in the presence of wrong sugar at biochemical and structural levels.

A previous study for ATP-dependent ligase from *Prochlorococcus marinus* reported post-step 3 structure of Pmar-Lig/nick DNA containing a canonical 3′-dC:G referring to a phosphodiester bond formation while AMP remained bound to the 5′-PO_4_ end of nick in the condition where the crystals were soaked in the buffer containing divalent metal ion (58). We compared this Pmar-Lig structure with our LIG1 structures and observed a very similar arrangement of the DNA after phosphodiester bond formation (Fig. S9). This structural overlay demonstrated that the active site amino acids of Pmar-Lig structure are highly closer to the active site amino acids of the LIG1 step 2 structure of 3′-dG:C (Fig. S9*A*). In addition, the amino acids in the LIG1 step 3 structures of 3′-rA:T and 3′-rG:C clash with the AMP, suggesting that the AMP in our structures is mostly released (Fig. S9*B*).

Although they share the highly conserved catalytic core and active site residues (31), we observed a difference in the ligation efficiency of LIG1 *versus* LIG3α for ribonucleotide-containing nicks. Future structure/function studies with LIG3α will be aimed at untangling the impact of improper sugar on the ligation reaction to gain a deeper understanding of how fidelity of nick sealing by other human DNA ligases is ensured to maintain genome integrity at the final step of DNA repair pathways when ribonucleotides embedded into genomic DNA due to their misincorporation by DNA pols. Previous studies demonstrated that LIG1 attempts to ligate the RNA-DNA lesion following RNase H2 cleavage, which results in the formation of abortive ligation products with 5′-adenylated-ribonucleotide that can be resolved by Aprataxin (12). Despite the fact that LIG1 is involved in the RER process, further structure/function studies are also required to elucidate how LIG1 surveils the RNA-DNA junctions harboring a 5′-ribonucleotide at atomic resolution.

## Experimental procedures

### Protein purifications

Human DNA ligase I (LIG1) proteins with 6x-his tag (pET-24b), the C-terminal (▵261) wild-type and E346A/E592A (EE/AA) mutant, were purified as described before (59, 60, 61, 62, 63, 64, 65). Briefly, the cells were overexpressed in Rosetta (DE3) *E. coli* cells in Terrific Broth (TB) media with kanamycin (50 μgml^−1^) and chloramphenicol (34 μgml^−1^) at 37 °C. The cells were induced with 0.5 mM isopropyl β-D-thiogalactoside (IPTG) when the OD_600_ was reached to 1.0, and the overexpression was continued for overnight at 28 °C. Cells were collected in the lysis buffer containing 50 mM Tris-HCl (pH 7.0), 500 mM NaCl, 20 mM imidazole, 10% glycerol, 1 mM phenylmethylsulfonyl fluoride (PMSF), and an EDTA-free protease inhibitor cocktail tablet by sonication at 4 °C. The cell lysate was pelleted at 31,000*g* for 1 h at 4 °C. LIG1 proteins were purified by HisTrap HP column with an increasing imidazole concentration (20–300 mM) after being equilibrated in the binding buffer containing 50 mM Tris-HCl (pH 7.0), 500 mM NaCl, 20 mM imidazole, and 10% glycerol at 4 °C. The collected fractions were subsequently loaded onto the HiTrap Heparin column that was equilibrated with the binding buffer containing 50 mM Tris-HCl (pH 7.0), 50 mM NaCl, 1.0 mM EDTA, and 10% glycerol, and then eluted with a linear gradient of NaCl up to 1 M. LIG1 proteins were further purified by Superdex 200 10/300 column in the buffer containing 20 mM Tris-HCl (pH 7.0), 200 mM NaCl, and 1 mM Dithiothreitol (DTT). Human wild-type full-length (1–922 amino acids) DNA ligase IIIα (LIG3α) with 6x-his tag (pET-29a) was overexpressed in BL21(DE3) *E. coli* cells in Luria-Bertani (LB) media at 37 °C for 8 h and induced with 0.5 mM IPTG. The protein overexpression was continued overnight at 28 °C. The cells were harvested, lysed at 4 °C, and then clarified as described above. LIG3α protein was purified by HisTrap HP column with an increasing imidazole gradient (20–300 mM) elution at 4 °C. The collected fractions were then further purified by Superdex 200 increase 10/300 column in the buffer containing 50 mM Tris-HCl (pH 7.0), 500 mM NaCl, glycerol 5%, and 1 mM DTT. All proteins were concentrated and stored at −80 °C. Protein quality was evaluated by running on 10% SDS-PAGE gel and the protein concentration was determined by the A280.

### Crystallization and structure determination

LIG1 C-terminal (▵261) EE/AA mutant (LIG1^EE/AA^) protein was used for the ligase crystallization (51). LIG1^EE/AA^ protein was mixed with nick DNA substrates containing 3′-rA:T, 3′-rG:C, and 3′-dG:C (Table S3). LIG1 (at 27 mgml^-1^)/DNA complex solution was prepared in the buffer containing 20 mM Tris-HCl (pH 7.0), 200 mM NaCl, 1 mM DTT, 1 mM EDTA, and 1 mM ATP at 1.4:1 DNA:protein molar ratio and then mixed with 1 μl reservoir solution. LIG1-nick DNA complex crystals were grown at 20 °C using the hanging drop method, harvested, and submerged in cryoprotectant solution containing reservoir solution mixed with glycerol to a final concentration of 20% glycerol before being flash cooled in liquid nitrogen for data collection (HKL Research, Inc). LIG1 crystals were obtained and kept at 100 °K during X-ray diffraction data collection using the beamline CHESS-7B2. LIG1 crystals for nick DNA containing canonical and ribonucleotide-containing ends were flash-frozen on different days as presented in Table S4. All structures were solved by the molecular replacement method using PHASER with PDB entry 7SUM as a search model (66). Iterative rounds of the model building were performed in COOT and the final models were refined with PHENIX (67, 68, 69). 3DNA was used for sugar pucker analysis (70). All structural images were drawn using PyMOL (The PyMOL Molecular Graphics System, V0.99, Schrödinger, LLC). Detailed crystallographic statistics are provided in Table 1.

### DNA ligation assays

DNA ligation assays were performed as reported (59, 60, 61, 62, 63, 64, 65) to evaluate the ligation efficiency of LIG1 (wild-type and EE/AA mutant) and LIG3α (Scheme S2). We used the nick DNA substrates containing a single ribonucleotide; 3′-rA:T, 3′-rC:G, 3′-rG:C; canonical base pairs 3′-dA:T, 3′-dC:G, 3′-dG:C; or mismatches 3′-dA:C and 3′-dG:T (Table S5). The ligation reaction containing 50 mM Tris-HCl (pH: 7.5), 100 mM KCl, 10 mM MgCl_2_, 1 mM ATP, 1 mM DTT, 100 μgml^−1^ BSA, 1% glycerol, and nick DNA substrate (500 nM) was initiated by the addition of DNA ligase (100 nM). The reaction samples were incubated at 37 °C, stopped by quenching with an equal amount of the buffer containing 95% formamide, 20 mM EDTA, 0.02% bromophenol blue, and 0.02% xylene cyanol, and collected at the time points indicated in the figure legends. The reaction products were then separated by electrophoresis on an 18% denaturing polyacrylamide gel. The gels were scanned with a Typhoon Phosphor Imager (Amersham Typhoon RGB), and the data were analyzed using ImageQuant software. The ligation assays were performed similarly in the absence of Mg^2+^.

### Data availability

Atomic coordinates and structure factors for the reported crystal structures of LIG1 have been deposited in the RCSB Protein Data Bank under accession numbers 3′-rA:T (8VDT, 8VZM), 3′-rG:C (8VDS, 8VZL), 3′-dG:C (8VDN). All data are contained within the manuscript. Further information and requests of materials used in this research are available from the authors upon reasonable request and should be directed to Dr Melike Çağlayan (caglayanm@ufl.edu).

## Supporting information

This article contains supporting information.

## Conflict of interest

The authors declare that they have no conflicts of interest with the contents of this article.

## Supporting information

Supplemental Figures 1–9, Tables 1–5 and Schemes 1–3

## References

[1] Klein H.L. (2017). Genome instabilities arising from ribonucleotides in DNA. DNA Repair.

[2] Potenski C.J., Klein H.L. (2014). How the misincorporation of ribonucleotides into genomic DNA can be both harmful and helpful to cells. Nucleic Acids Res..

[3] Nick McElhinny S.A., Kumar D., Clark A.B., Watt D.L., Watts B.E., Lundstrom E.B. (2010). Genome instability due to ribonucleotide incorporation into DNA. Nat. Chem. Biol..

[4] Yao N.Y., Schroeder J.W., Yurieva O., Simmons L.A., O’Donnell M.E. (2013). Cost of rNTP/dNTP pool imbalance at the replication fork. Proc. Natl. Acad. Sci. U. S. A..

[5] Caldecott K.W.R. (2014). Ribose-an internal threat to DNA. Science.

[6] Hovatter K.R., Martinson H.G. (1987). Ribonucleotide-induced helical alteration in DNA prevents nucleosome formation. Proc. Natl. Acad. Sci. U. S. A..

[7] Conover H.N., Lujan S.A., Chapman M.J., Cornello D.A., Sharif R., Williams J.S. (2015). Stimulation of chromosomal re-arrangements by ribonucleotides. Genetics.

[8] Williams J.S., Kunkel T.A. (2014). Ribonucleotides in DNA: origins, repair and consequences. DNA Repair.

[9] Reijns M.A., Rabe B., Rigby R.E., Mill P., Astell K.R., Lettice L.A. (2012). Enzymatic removal of ribonucleotides from DNA is essential for mammalian genome integrity and development. Cell.

[10] Sparks J.L., Chon H., Cerritelli S.M., Kunkel T.A., Johansson E., Crouch R.J. (2012). RNase H2-initiated ribonucleotide excision repair. Mol. Cell.

[11] Hiller B., Achleitner M., Glage S., Naumann R., Behrendt R., Roers A. (2012). Mammalian RNase H2 removes ribonucleotides from DNA to maintain genome integrity. J. Exp. Med..

[12] Tumbale P., Williams J.S., Schellenberg M.J., Kunkel T.A., Williams R.S. (2014). Aprataxin resolves adenylated RNA-DNA junctions to maintain genome integrity. Nature.

[13] Williams J.S., Clausen S.A., Lujan I., Marjavaara A.B., Clark P.M., Burgers A. (2015). Evidence that processing of ribonucleotides in DNA by topoisomerase 1 is leasing-strand specific. Nat. Struc. Mol. Biol..

[14] Chon H., Sparks J.L., Rychlik M., Nowotny M., Burgers P.M., Crouch R.J. (2013). RNase H2 roles in genome integrity revealed by unlinking its activities. Nucleic Acids Res..

[15] Crow Y.J., Leitch A., Hayward B.E., Garner A., Parmar R., Griffith E. (2006). Mutations in genes encoding ribonuclease H2 subunits cause Aicardi-Goutieres syndrome and mimic congenital viral brain infection. Nat. Genet..

[16] Williams J.S., Lujan S.A., Kunkel T.A. (2016). Processing ribonucleotides incorporated during eukaryotic DNA replication. Nat. Rev. Mol. Cell Biol..

[17] Joyce C.M. (1997). Choosing the right sugar: how polymerases select a nucleotide substrate. Proc. Natl. Acad. Sci. U. S. A..

[18] Brown J.A., Suo Z. (2011). Unlocking the sugar ‘steric gate’ of DNA polymerases. Biochemistry.

[19] Brown J.A., Fiala K.A., Fowler J.D., Sherrer S.M., Newmister S.A., Duym W.A. (2010). A novel mechanism of sugar selection utilized by a human X-family DNA polymerase. J. Mol. Biol..

[20] Clausen A.R., Zhang S., Burgers P.N., Lee M.Y., Kunkel T.A. (2013). Ribonucleotide incorporation, proofreading and bypass by human DNA polymerase delta. DNA Repair.

[21] Nick McElhinny S.A., Watts B.E., Kumar D., Watt D.L., Lundstrom E., Burgers P.M. (2010). Abundant ribonucleotide incorporation into DNA by yeast replicative polymerases. Proc. Natl. Acad. Sci. U. S. A..

[22] Clausen A.R., Murray M.S., Passer A.R., Pedersen L.C., Kunkel T.A. (2013). Structure-function analysis of ribonucleotide bypass by B family DNA replicases. Proc. Natl. Acad. Sci. U. S. A..

[23] Gosavi R.A., Moon A.F., Kunkel T.A., Pedersen L.C., Bebenek K. (2012). The catalytic cycle for ribonucleotide incorporation by human DNA pol lambda. Nucleic Acids Res..

[24] Moon A.F., Pryor J.M., Ramsden D.A., Kunkel T.A., Bebenek K., Pedersen L.C. (2017). Structural accommodation of ribonucleotide incorporation by the DNA repair enzyme polymerase μ. Nucleic Acids Res..

[25] Ruiz J.F., Juarez R., Garcia-Diaz M., Terrados G., Picher A.J., Gonzalez-Barrera S. (2014). Lack of sugar discrimination by human pol μ requires a single glycine residue. Nucleic Acids Res..

[26] Cavanaugh N.A., Beard W.A., Wilson S.H. (2010). DNA polymerase β ribonucleotide discrimination. J. Biol. Chem..

[27] Kasiviswanathan R., Copeland W.C. (2011). Ribonucleotide discrimination and reverse transcription by the human mitochondrial DNA polymerase. J. Biol. Chem..

[28] Donigan K.A., McLenigan M.P., Yang W., Goodman M.F., Woodgate R. (2014). The steric gate of DNA polymerase iota regulates ribonucleotide incorporation and deoxyribonucleotide fidelity. J. Biol. Chem..

[29] DeLucia A.M., Grindley N.D., Joyce C.M. (2003). An error-prone family Y DNA polymerase (DinB homolog from Sulfolobus solfataricus) uses a ‘steric gate' residue for dis- crimination against ribonucleotides. Nucleic Acids Res..

[30] Vaisman A., Woodgate R. (2018). Ribonucleotide discrimination by translesion synthesis DNA polymerases. Crit. Rev. Biochem. Mol. Biol..

[31] Ellenberger T., Tomkinson A.E. (2008). Eukaryotic DNA ligases: structural and functional insights. Annu. Rev. Biochem..

[32] Timson D.J., Singleton M.R., Wigley D.B. (2000). DNA ligases in the repair and replication of DNA. Mutat. Res..

[33] Tomkinson A.E., Vijayakumar S., Pascal J.M., Ellenberger T. (2006). DNA ligases: structure, reaction mechanism, and function. Chem. Rev..

[34] Doherty A.J., Suh S.W. (2000). Structural and mechanistic conservation in DNA ligases. Nucleic Acids Res..

[35] Çağlayan M. (2019). Interplay between DNA polymerases and DNA ligases: influence on substrate channeling and the fidelity of DNA ligation. J. Mol. Biol..

[36] Tomkinson A.E., Tottyt N.F., Ginsburg M., Lindahl T. (1991). Location of the active site for enzyme-adenylate formation in DNA ligases. Proc. Natl. Acad. Sci. U. S. A..

[37] Cherepanov A.V., De Vries S. (2002). Dynamic mechanism of nick recognition by DNA ligase. Eur. J. Biochem..

[38] Yang S.-W., Chans J.Y.H. (1992). Analysis of the Formation of AMP-DNA intermediate and the successive reaction by human DNA ligases I and II. J. Biol. Chem..

[39] Dickson K.S., Burns C.M., Richardson J.P. (2000). Determination of the free-energy change for repair of a DNA phosphodiester bond. J. Biol. Chem..

[40] Petrini J.H., Xiao Y., Weaver D.T. (1995). DNA ligase I mediates essential functions in mammalian cells. Mol. Cell. Biol..

[41] Sun D., Urrabaz R., Nguyen M., Marty J., Stringer S., Cruz E. (2001). Elevated expression of DNA ligase I in human cancers. Clin. Cancer Res..

[42] Han L., Masani S., Hsleh C., Yu K. (2014). DNA ligase I is not essential for mammalian cell viability. Cell Rep..

[43] Levin D.S., McKenna A.E., Motycka T.A., Matsumoto Y., Tomkinson A.E. (2000). Interaction between PCNA and DNA ligase I is critical for joining of Okazaki fragments and long-patch base-excision repair. Brief Comm..

[44] Tom S., Henricksen L.A., Park M.S., Bambara R.A. (2001). DNA ligase I and proliferating cell nuclear antigen form a functional complex. J. Biol. Chem..

[45] Song W., Pascal J.M., Ellenberger T., Tomkinson A.E. (2009). The DNA binding domain of human DNA ligase I interacts with both nicked DNA and the DNA sliding clamps, PCNA and hRad9- hRad1-hHus1. DNA Repair.

[46] Wang W., Boltz L.A., Sancar A., Bambara R.A. (2006). Mechanism of stimulation of human DNA ligase I by the Rad9- Rad1-Hus1 checkpoint complex. J. Biol. Chem..

[47] Pascal J.M., O’Brien P.J., Tomkinson A.E., Ellenberger T. (2004). Human DNA ligase I completely encircles and partially unwinds nicked DNA. Nature.

[48] Tumbale P.P., Jurkiw T.J., Schellenberg M.J., Riccio A.A., O’Brien P.J., Williams R.S. (2019). Two-tiered enforcement of high-fidelity DNA ligation. Nat. Commun..

[49] Williams J.S., Tumbale P.P., Arana M.E., Rana J.A., Williams R.S., Kunkel T.A. (2021). High-fidelity DNA ligation enforces accurate Okazaki fragment maturation during DNA replication. Nat. Commun..

[50] Jurkiw T.J., Tumbale P.P., Schellenberg M.J., Cunningham-Rundles C., Williams R.S., O’Brien P.J. (2021). LIG1 syndrome mutations remodel a cooperative network of ligand binding interactions to compromise ligation efficiency. Nucleic Acids Res..

[51] Tang Q., Gulkis M., McKenna R., Çağlayan M. (2022). Structures of LIG1 that engage with mutagenic mismatches inserted by polβ in base excision repair. Nat. Commun..

[52] Luo J., Barany F. (1996). Identification of essential residues in *Thermus thermophilus* DNA ligase. Nucleic Acids Res..

[53] Tomkinson A.E., Tappe N.J., Friedberg E.C. (1992). DNA ligase I from *Saccharomyces cerevisiae*: physical and biochemical characterization of the CDC9 gene product. Biochemistry.

[54] Sriskanda V., Shuman S. (1998). Specificity and fidelity of strand joining by *Chlorella virus* DNA ligase. Nucleic Acids Res..

[55] Shuman S. (1995). Vaccinia virus DNA ligase: specificity, fidelity, and inhibition. Biochemistry.

[56] Çağlayan M. (2020). The ligation of pol β mismatch insertion products governs the formation of promutagenic base excision DNA repair intermediates. Nucleic Acids Res..

[57] Dejaegere A.P., Case D.A. (1998). Density functional study of ribose and deoxyribose chemical shifts. J. Phys. Chem..

[58] Williamson A., Leiros H.K.S. (2019). Structural intermediates of a DNA-ligase complex illuminate the role of the catalytic metal ion and mechanism of phosphodiester bond formation. Nucleic Acids Res..

[59] çağlayan M., Batra V.K., Sassa A., Prasad R., Wilson S.H. (2014). Role of polymerase β in complementing aprataxin deficiency during abasic-site base excision repair. Nat. Struct. Mol. Biol..

[60] Çağlayan M., Prasad R., Krasich R., Longley M.J., Kadoda K., Tsuda M. (2015). Complementation of aprataxin deficiency by base excision repair enzymes. Nucleic Acids Res..

[61] Çağlayan M., Prasad R., Krasich R., Longley M.J., Kadoda K., Tsuda M. (2017). Complementation of aprataxin deficiency by base excision repair enzymes in mitochondrial extracts. Nucleic Acids Res..

[62] Çağlayan M., Horton J.K., Dai D.P., Stefanick D.F., Wilson S.H. (2017). Oxidized nucleotide insertion by pol β confounds ligation during base excision repair. Nat. Commun..

[63] Çağlayan M. (2020). Pol β gap filling, DNA ligation and substrate-product channeling during base excision repair opposite oxidized 5-methylcytosine modifications. DNA Repair.

[64] Tang Q., Kamble P., Çağlayan M. (2020). DNA ligase I variants fail in the ligation of mutagenic repair intermediates with mismatches and oxidative DNA damage. Mutagenesis.

[65] Kamble P., Hall K., Chandak M., Tang Q., Çağlayan M. (2021). DNA ligase I fidelity the mutagenic ligation of pol β oxidized and mismatch nucleotide insertion products in base excision repair. J. Biol. Chem..

[66] McCoy A.J., Grosse-Kunstleve R.W., Adams P.D., Winn M.D., Storoni L.C., Read R.J. (2007). Phaser crystallographic software. J. Appl. Crystallogr..

[67] Emsley P., Lohkamp B., Scott W.G., Cowtan K. (2010). Features and development of Coot. Acta Crystallogr. D. Biol. Crystallogr..

[68] Adams P.D., Afonine P.V., Bunkoczo G., Chen V.B., Davis I.W., Echold N. (2010). PHENIX: a comprehensive Python-based system for macromolecular structure solution. Acta Crystallogr. D. Biol. Crystallogr..

[69] Murshudov G.N., Skubak P., Lebedev A.A., Pannu N.S., Steiner R.A., Nicholls R.A. (2011). REFMAC5 for the refinement of macromolecular crystal structures. Acta Crystallogr. D. Biol. Crystallogr..

[70] Li S., Olson W.K., Lu X.J. (2019). Web 3DNA 2.0 for the analysis, visualization, and modeling of 3D nucleic acid structures. Nucleic Acids Res..

