# Supplementary material for: Structures of LIG1 provide a mechanistic basis for understanding a lack of sugar discrimination against a ribonucleotide at the 3'-end of nick DNA
Source: J Biol Chem. 2024 Mar 24;300(5):107216. doi: 10.1016/j.jbc.2024.107216 (PMC11035063; doi:10.1016/j.jbc.2024.107216)
Supplement: Supplemental Figures 1–9, Tables 1–5 and Schemes 1–3 [file mmc1.pdf]

Structures of LIG1 provide a mechanistic basis for understanding a lack of sugar discrimination against a ribonucleotide at the 3'-end of nick DNA

Kanal Elamparithi Balu, Mitchell Gulkis, Danah Almohdar and Melike Çağlayan*

Department of Biochemistry and Molecular Biology, University of Florida, Gainesville, FL 32610, USA

*To whom correspondence should be addressed. Tel.: +1 352-294-8383; E-mail: caglayanm@ufl.edu

Running Title: Inability to exclude ribonucleotide at nick by DNA ligase 1

Supplementary Figures 1-9

Supplementary Tables 1-5

Supplementary Schemes 1-3

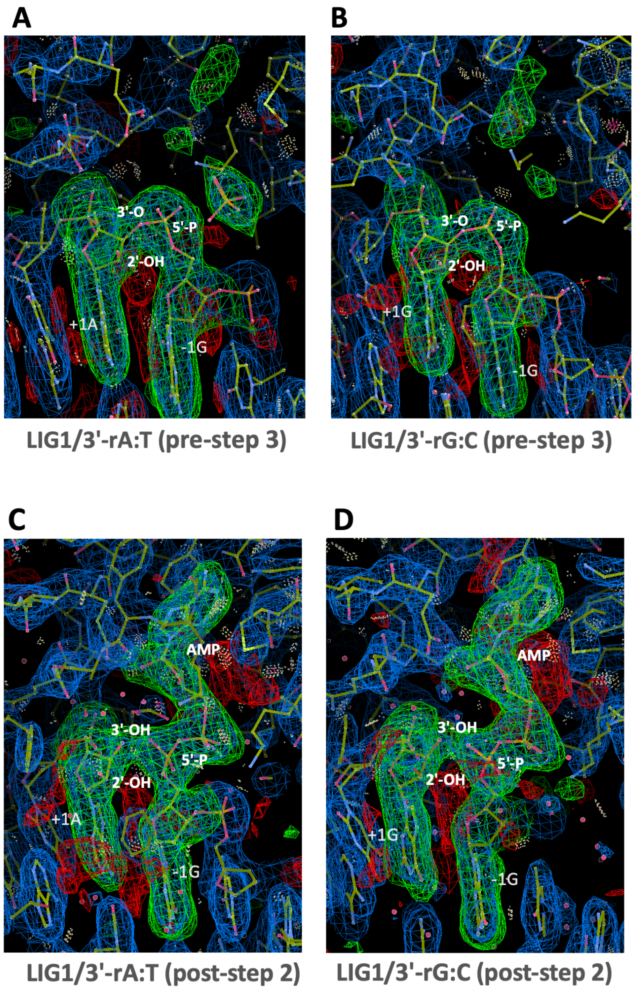

Supplementary Figure 1. LIG1/RNA-DNA heteroduplex structures show the differences in the position of AMP at the last two steps of the ligation reaction. Structures of LIG1 in complex with nick DNA containing 3'-ribonucleotide at the nick were shown for the post-step 3 (**A-B**) and pre-step 3 (**C-D**) structures of 3'-rA:T and 3'-rG:C. Simulated annealing omit maps (Fo-Fc) of the AMP are contoured at 3σ. The map for AMP is incomplete in the LIG1 structures solved in the pre-step 3 of the ligation reaction.

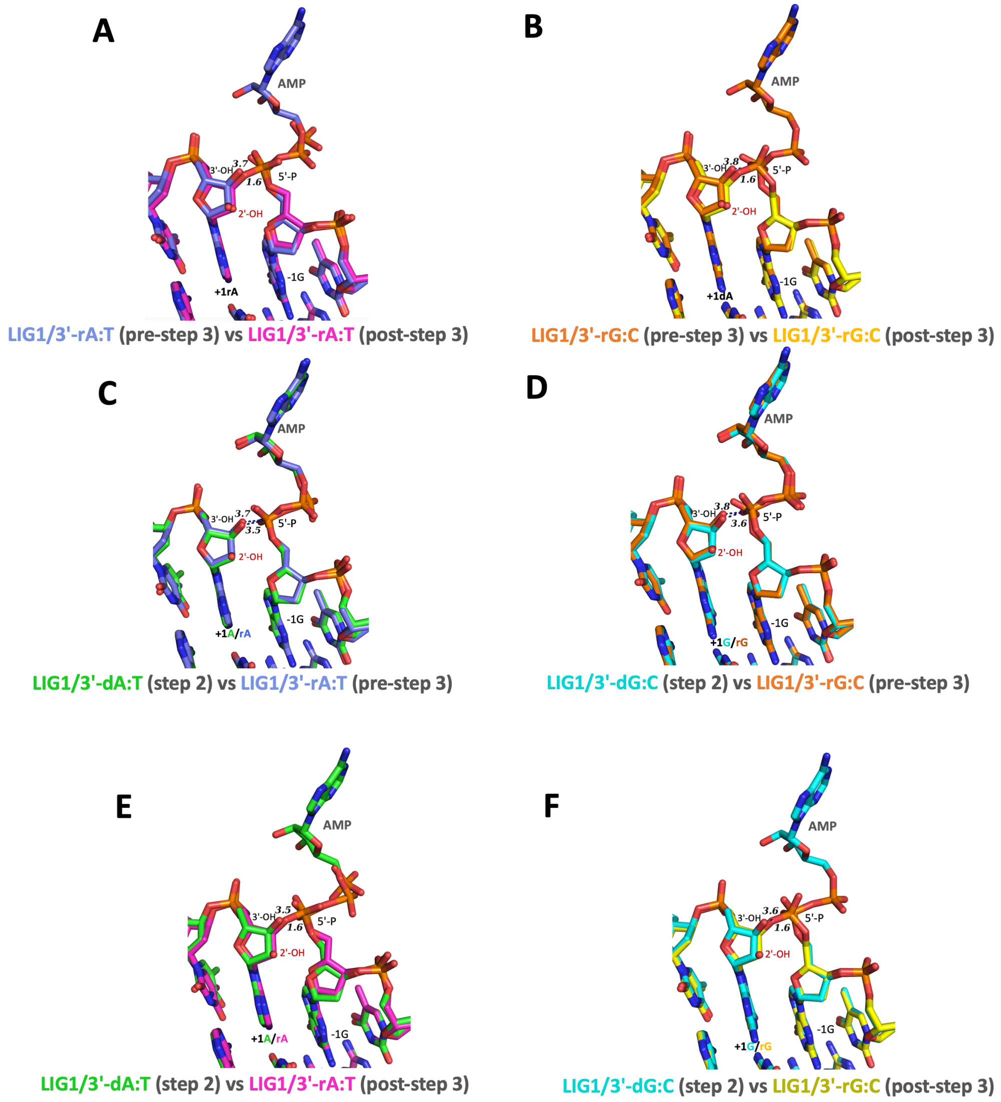

Supplementary Figure 2. Overlay of LIG1 structures in complex with nick DNA duplexes captured at difference steps of the ligation reaction. (A-F) Superimpositions of LIG1 structures are shown for the nick DNA complexes containing 3'-deoxyribonucleotide (step 2) *versus* 3'-ribonucleotide (pre- and post-step 3) for 3'-rA/dA:T and 3'-rG/dG:C.

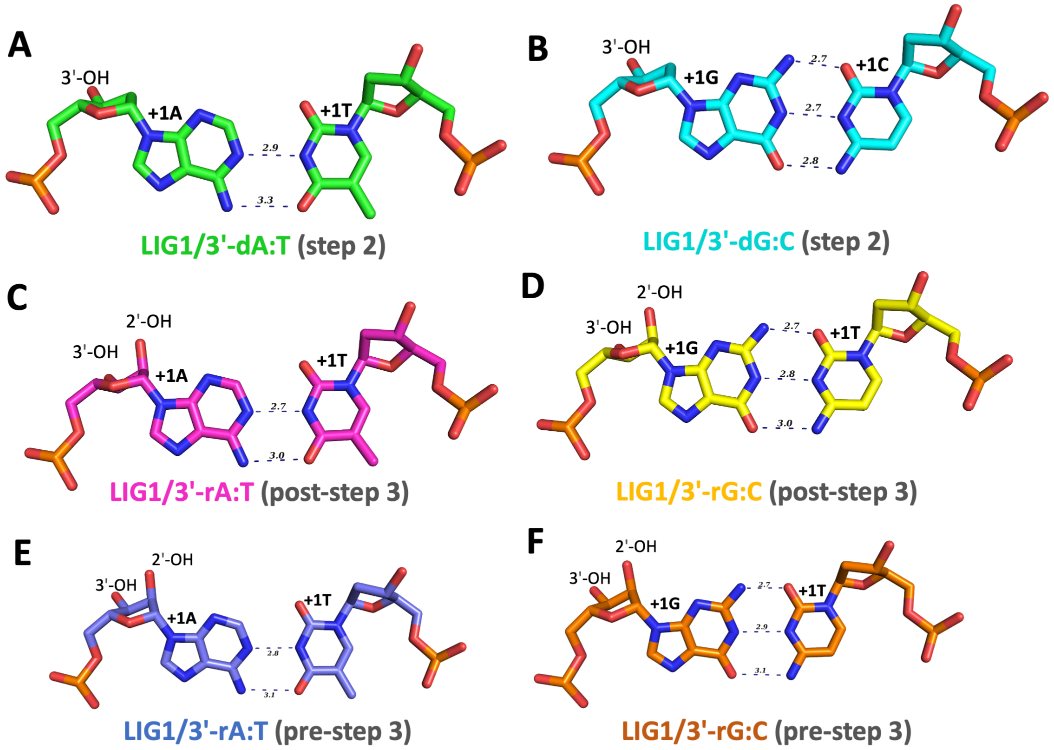

Supplementary Figure 3. Base-pairing architecture of LIG1 structures. Watson-Crick base pairing between 3'- and 5'-terminus of the nick DNA are shown for LIG1 structures captured at the step 2 of 3'-dA:T and 3'-dG:C (**A-B**), the post-step 3 (**C-D**) and pre-step 3 (**E-F**) of 3'-rA:T and 3'-rG:C.

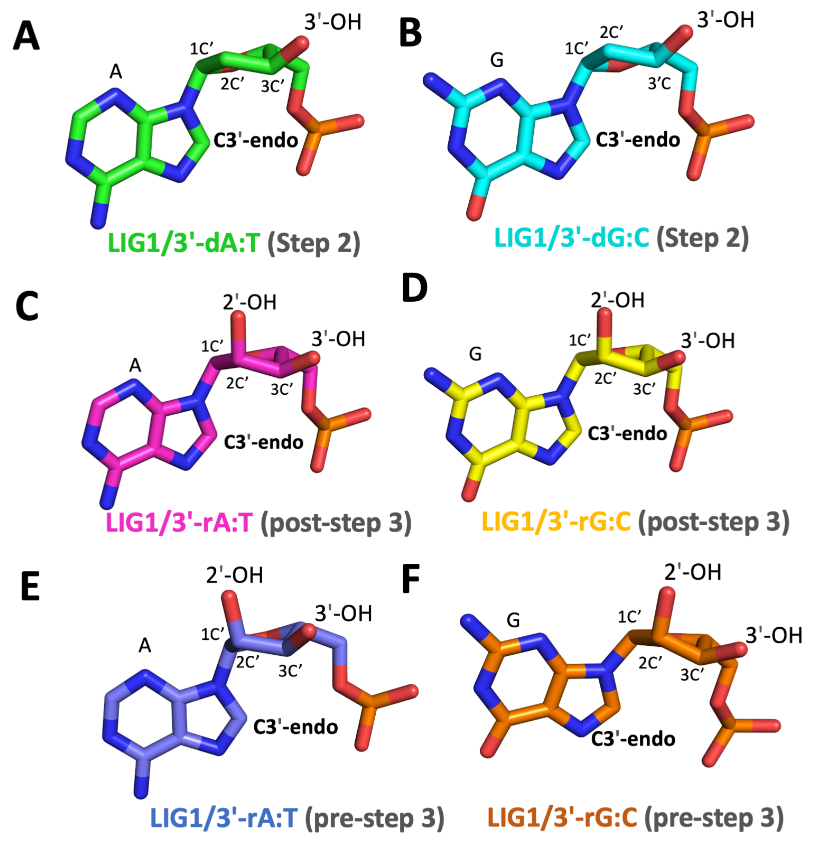

Supplementary Figure 4. Sugar pucker analyses of LIG1 structures solved in this study. 3'-end sugar pucker analyses of LIG1 structures are presented for LIG1 structures captured at the step 2 of 3'-dA:T and 3'-dG:C (**A-B**), the post-step 3 (**C-D**) and pre-step 3 (**E-F**) of 3'-rA:T and 3'-rG:C.

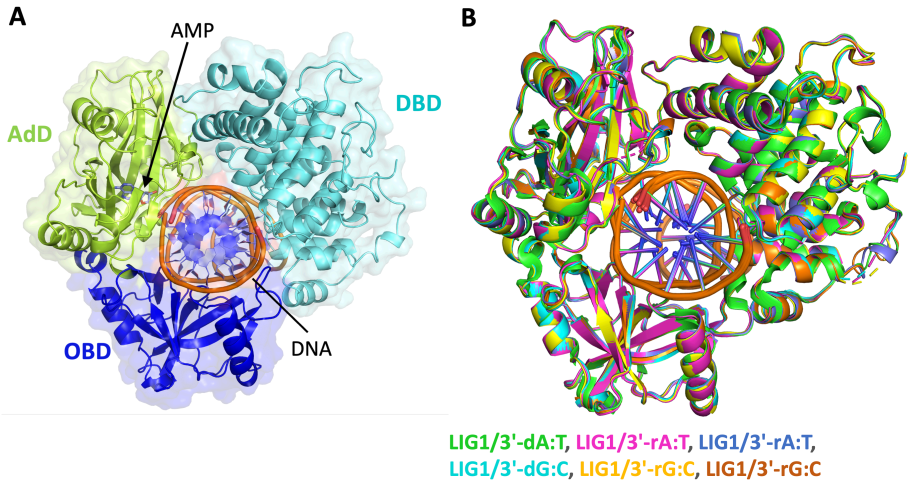

Supplementary Figure 5. X-ray structures of LIG1-nick DNA complexes. (A) LIG1-AMP complex shows the catalytic core consisting of Adenylation (AdD, green), DNA-binding (DBD, cyan), and Oligonucleotide-binding (OBD, blue) domains that encircle the nick DNA substrate (orange). **(B)** The superimposition of all six LIG1 structures in the present study demonstrate a global conformation that the catalytic core adopts with 3'-deoxyribonucleotide *versus* 3'-ribonucleotide at the nick.

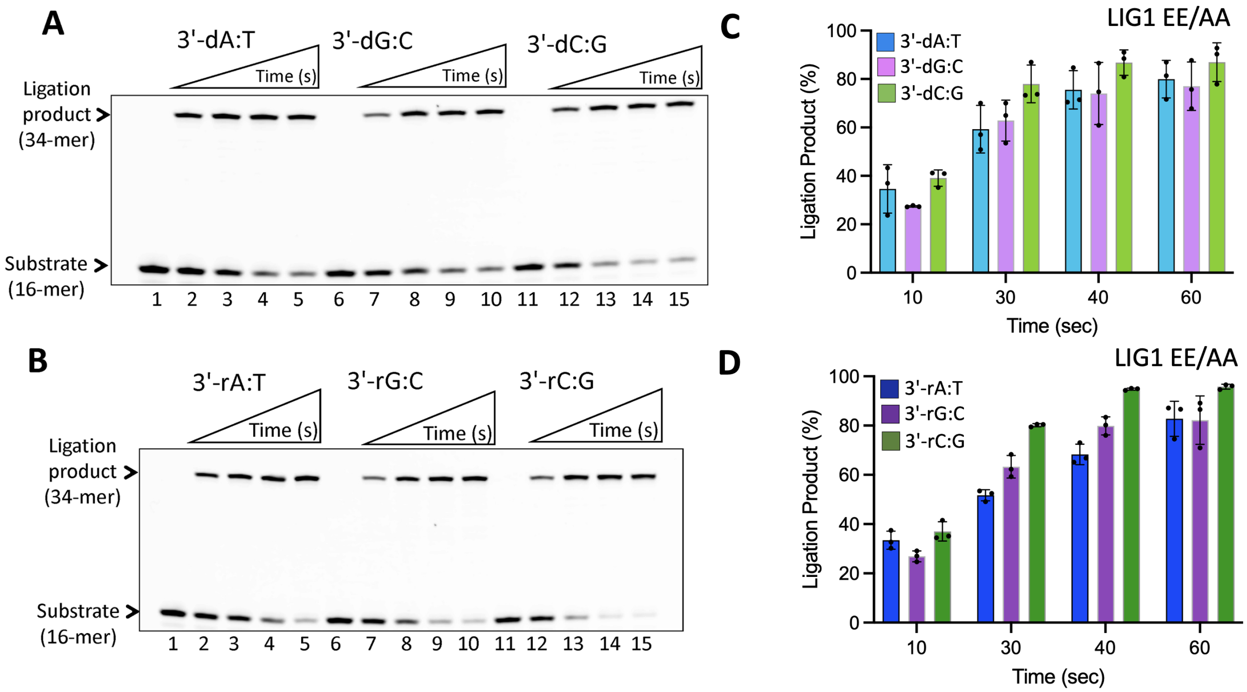

Supplementary Figure 6. Ligation of the nick DNA substrates with 3'-deoxyribonucleotides *versus* 3'-ribonucleotides by LIG1 EE/AA low-fidelity mutant. (A) Lanes 1, 6 and 11 are the negative enzyme controls of the nick DNA substrates and lanes 2-5, 7-10, and 12-15 are the ligation products in the presence of 3'-dA:T, 3'-dG:C, and 3'-dC:G, respectively, and correspond to time points of 10, 30, 40, and 60 sec. **(B)** Lanes 1, 6 and 11 are the negative enzyme controls of the nick DNA substrates and lanes 2-5, 7-10, and 12-15 are the ligation products in the presence of 3'-rA:T, 3'-rG:C, and 3'-rC:G, respectively, and correspond to time points of 10, 30, 40, and 60 sec. **(C-D)** Graphs show time-dependent changes in the amount of ligation products and the data represent the average from three independent experiments ± SD.

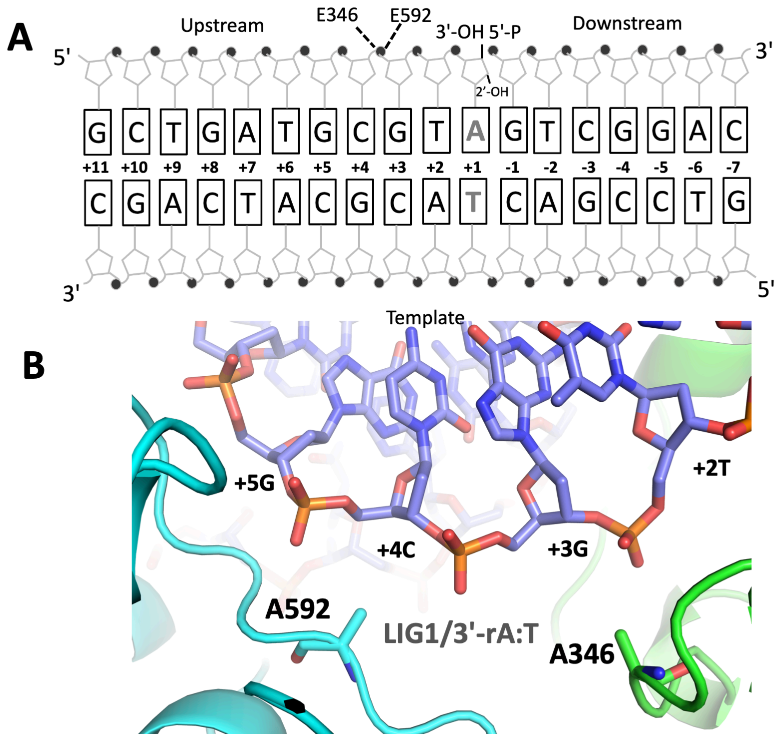

Supplementary Figure 7. Structures of LIG1$^{EE/AA}$ demonstrate the position of the high-fidelity site, EE/AA. (A) Schematic view of nick DNA showing the amino acids E346 and E592 that are identified as high-fidelity and Mg^{2+} binding site. **(B)** The mutations at this side (E346/A346 and E592/A592, referred as EE/AA) are shown in the LIG1 structures.

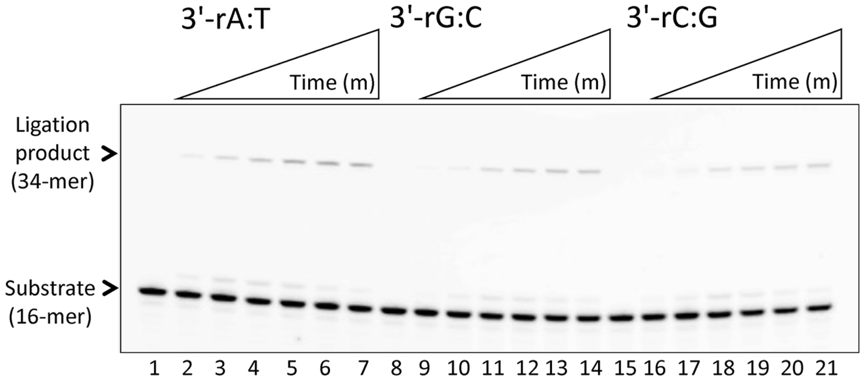

Supplementary Figure 8. Ligation of the nick DNA substrates with 3'-ribonucleotides in the absence of Mg^{2+} by LIG1. (A) Lanes 1, 8 and 15 are the negative enzyme controls of the nick DNA substrates, and lanes 2-7, 9-14, and 16-21 are the ligation products in the presence of 3'-rA:T, 3'-rG:C, and 3'-rC:G, respectively, and correspond to time points of 0.5, 1, 3, 5, 8, and 10 min.

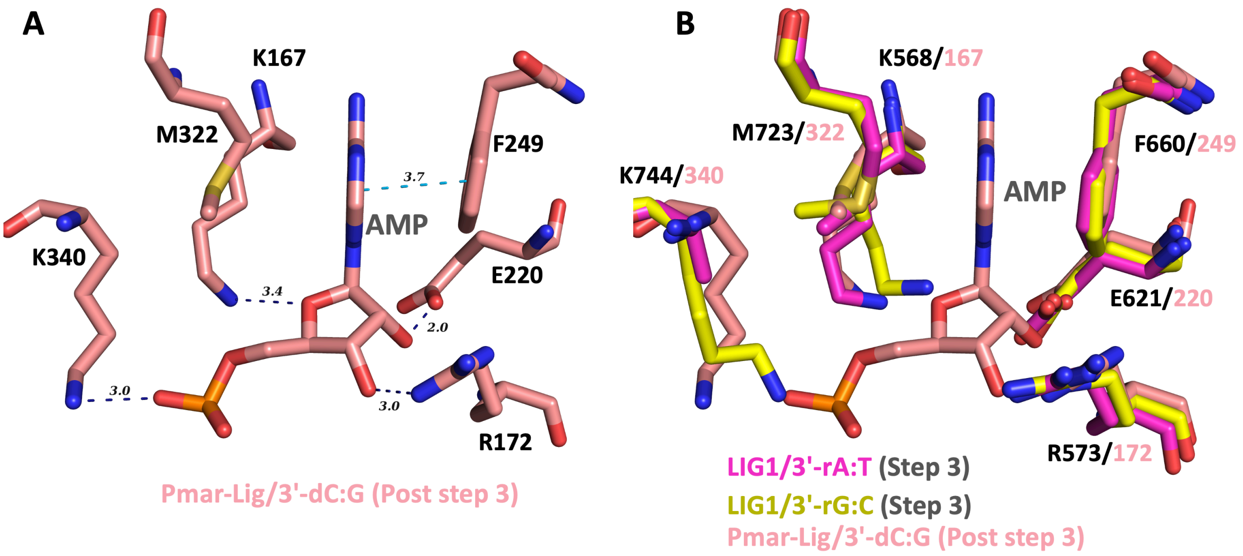

Supplementary Figure 9. Comparison of Pmar-Lig and LIG1 structures. The post step 3 structure of ATP-dependent ligase from *Prochlorococcus marinus* (Pmar-Lig) is used (PDB: 6RAU) to compare with LIG1 step 2 and step 3 structures of the present study. (**A**) The overlay for the active site amino acid residues of Pmar-Lig shares positions of the step 2 structure of LIG1/3'-dG:C. (**B**) The superposition for post step 3 structure of Pmar-Lig and step 3 structures of LIG1/3'-rA:T and LIG1/3'-rG:C shows that the active site amino acids of LIG1 structures clashes with AMP of Pmar-Lig structure. RMSD between Pmar-Lig and our step 2 structure is ~0.5A with respect to AMP. The deviation in the sidechains is calculated to be ~0. 2A.

DNA ligase 1	DNA	Ligation Step	Reference
LIG1^{WT}	3'-ddC:G	Step 2	48
LIG1^{WT}	3'-ddC:G+Mg^{2+}	Step 2	49
LIG1^{WT}	3'-dC:G	Step 2	49
LIG1$^{EE/AA}$	3'-dC:G	Step 2	49
LIG1$^{EE/AA}$	3'-8oxodG:A	Step 2	49
LIG1$^{EE/AA}$	3'-dC:G	Step 2	49
LIG1$^{EE/AA}$	Bulged DNA	Step 2	50
LIG1^{R641L}	3'-dC:G	Step 2	51
LIG1^{R771W}	3'-dC:G	Step 2	51
LIG1$^{EE/AA}$	3'-dA:T	Step 2	52
LIG1$^{EE/AA}$	3'-dG:T	Step 2	52
LIG1$^{EE/AA}$	3'-dA:C	Step 1	52
LIG1$^{EE/AA}$	3'-dG:C	Step 2	Present study
LIG1$^{EE/AA}$	3'-rA:T	Pre-step 3	Present study
LIG1$^{EE/AA}$	3'-rG:C	Pre-step 3	Present study
LIG1$^{EE/AA}$	3'-rA:T	Post-step 3	Present study
LIG1$^{EE/AA}$	3'-rG:C	Post-step 3	Present study

Supplementary Table 1. The list of LIG1 structures solved previously and presented in this study.

**RMSD Å**	LIG1$^{EE/AA}$ 3'-dA:T	LIG1$^{EE/AA}$ 3'-rA:T	LIG1$^{EE/AA}$ 3'-dG:C	LIG1$^{EE/AA}$ 3'-rG:C	LIG1$^{EE/AA}$ 3'-dA:C	LIG1$^{EE/AA}$ 3'-dG:T
LIG1$^{EE/AA}$ 3'-dA:T		0.537	0.393	0.444	0.970	0.539
LIG1$^{EE/AA}$ 3'-rA:T			0.296	0.296	0.668	0.379
LIG1$^{EE/AA}$ 3'-dG:C				0.241	0.737	0.375
LIG1$^{EE/AA}$ 3'-rG:C					0.715	0.353
LIG1$^{EE/AA}$ 3'-dA:C						0.655

Supplementary Table 2. The root mean square deviation (RMSD) of LIG1 structures solved previously and presented in this study.

Oligonucleotide	Sequence (5'-3')
Template T	GTCCGACTACGCATCAGC
Template C	GTCCGACCACGCATCAGC
Upstream A (3'-rA)	GCTGATGCGT**A**
Upstream G (3'-rG)	GCTGATGCGT**G**
Upstream G (3'-dG)	GCTGATGCGT**G**
Downstream (5'-P)	P-GTCGGAC

Supplementary Table 3. Oligonucleotides used in LIG1 crystallization. Upstream oligonucleotides including a single ribonucleotide at the 3'-end (rA and rG), upstream oligonucleotide including a canonical base at the 3'-end (dG), downstream oligo with phosphate (P) at the 5'-end, and template oligonucleotides containing T or C on a template position were used to prepare the nick DNA substrates with 3'-ribonucleotides (3'-rA:T and 3'-rG:C) and 3'-deoxyribonucleotide (3'-dG:C) for LIG1 crystallizations. The base at template base position is underlined and the base position at the 3'-end of nick is shown in bold.

$LIG1^{EE/AA}$	Crystal conditions	Crystallization time (days)
3'-rA:T (pre-step 3)	100 mM MES (pH 6.7), 100 mM lithium acetate, and 16% (w/v) PEG3350	1
3'-rA:T (post-step 3)	100 mM MES (pH 6.7), 100 mM lithium acetate, and 16% (w/v) PEG3350	3
3'-dG:C (step 2)	100 mM MES (pH 6.1), 100 mM lithium acetate, and 10% (w/v) PEG3350	1
3'-rG:C (pre-step 3)	100 mM MES (pH 7.0), 100 mM lithium acetate, and 14% (w/v) PEG3350	1
3'-rG:C (post-step 3)	100 mM MES (pH 7.0), 100 mM lithium acetate, and 14% (w/v) PEG3350	3

Supplementary Table 4. Crystallization conditions of LIG1 structures solved in the study.

Nick DNA Substrates	Sequence
3'-dA:T	5'-CATGGGCGGCATGAACC**A**GAGGCCCATCCTCACC-3-FAM<br>3'-GTACCCGCCGTACTTGGTCTCCGGGTAGGAGTGG-5'
3'-dC:G	5'-CATGGGCGGCATGAACC**C**GAGGCCCATCCTCACC-3-FAM<br>3'-GTACCCGCCGTACTTGGGCTCCGGGTAGGAGTGG-5'
3'-dG:C	5'-CATGGGCGGCATGAACC**G**GAGGCCCATCCTCACC-3-FAM<br>3'-GTACCCGCCGTACTTGGCCTCCGGGTAGGAGTGG-5'
3'-dA:C	5'-CATGGGCGGCATGAACC**A**GAGGCCCATCCTCACC-3'-FAM<br>3'-GTACCCGCCGTACTTGGCCTCCGGGTAGGAGTGG-5'
3'-dG:T	5'-CATGGGCGGCATGAACC**G**GAGGCCCATCCTCACC-3'-FAM<br>3'-GTACCCGCCGTACTTGGTCTCCGGGTAGGAGTGG-5'
3'-rA:T	5'-CATGGGCGGCATGAACC^{r}**A**GAGGCCCATCCTCACC-3-FAM<br>3'-GTACCCGCCGTACTTGGTCTCCGGGTAGGAGTGG-5'
3'-rC:G	5'-CATGGGCGGCATGAACC^{r}**C**GAGGCCCATCCTCACC-3-FAM<br>3'-GTACCCGCCGTACTTGGGCTCCGGGTAGGAGTGG-5'
3'-rG:C	5'-CATGGGCGGCATGAACC^{r}**G**GAGGCCCATCCTCACC-3-FAM<br>3'-GTACCCGCCGTACTTGGCCTCCGGGTAGGAGTGG-5'

Supplementary Table 5. Nick DNA substrates used in ligation assays. FAM denotes a fluorescence tag and is located at the 3'-end of the nick DNA substrates. The base at 3'-end is shown as bold and the template base is underlined.

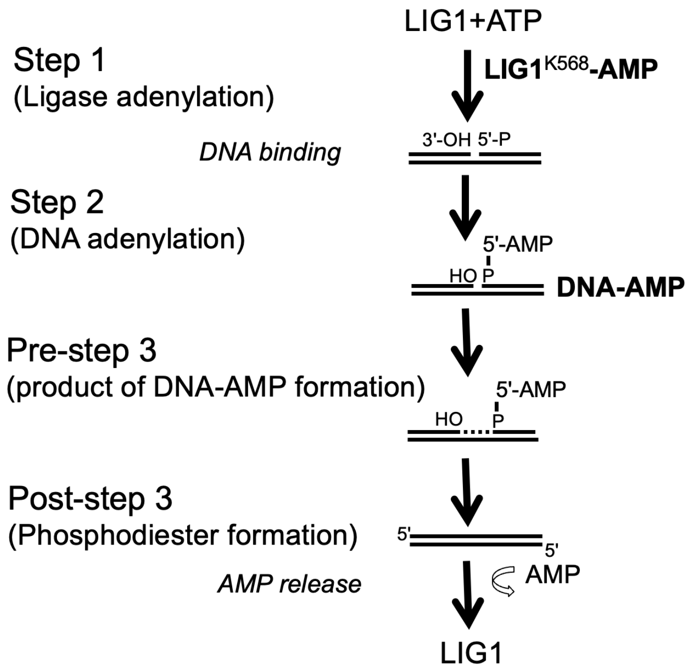

Supplementary Scheme 1. Three consecutive steps of DNA ligation reaction. In the first step, the ligase attacks the α-phosphate of ATP, which results in an adenylate (AMP) moiety being covalently linked to the ε-amino group of an active site lysine residue, (K568 of LIG1), resulting in the formation of the initial DNA ligase-AMP intermediate. The adenylated ligase then undergoes a conformational change which allows the enzyme to surveille the genome until a nick is encountered where DNA binding occurs. In the second step of the ligation reaction, the ligase catalyzes the transfer of AMP to the 5'-PO_4 end referring to DNA adenylation, which is followed by forming a 5'-5' phosphoanhydride high energy bond, leading to the formation the DNA-AMP product complex (referred to pre-step 3 in this study). Finally, in the third step (referred to post step 3 in this study), the ligase catalyzes in-line nucleophilic attack of the 3'-OH group onto the 5'-PO_4 creating a new phosphodiester bond, which is followed by displacing AMP as a leaving group.

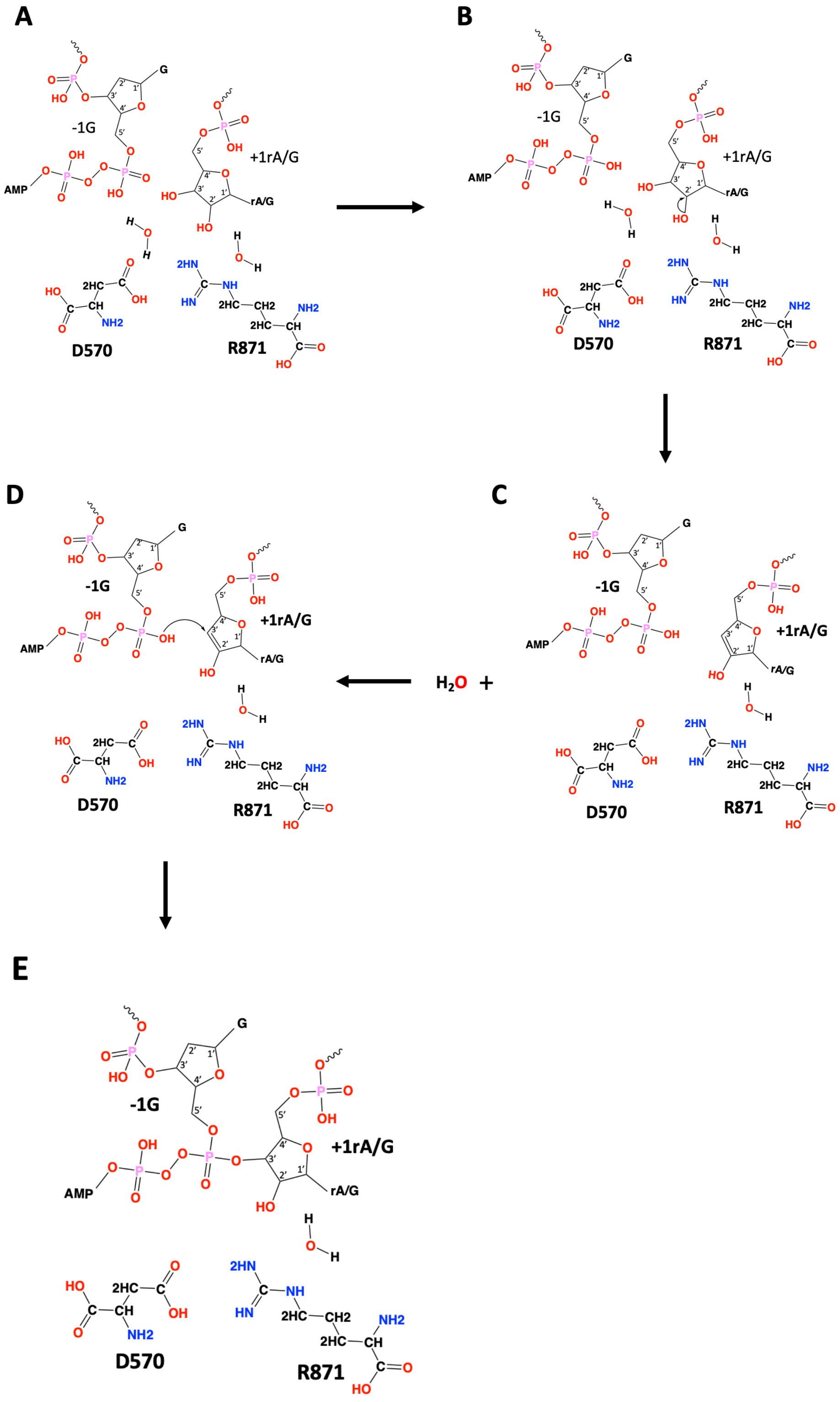
A
B
C
D
E
-1G
+1rA/G
AMP
rA/G
G
D570
R871
H2O +

Supplementary Scheme 2. Potential reaction leading to a lack of sugar discrimination at the 3'-end of nick by LIG1. (**A-E**) Based on the LIG1 pre- and post-step 3 structures, a phosphate diester bond could form by the condensation reaction between 3'-OH and 5'-PO_4 ends of the nick. In pre-step 3 structures (A), the water bridge between D570 and 3'-OH and the interaction between 2'-OH and the sidechain of R871 provide a possible chemical conformation for the condensation reaction. The interaction of R871 creates electron localization at 2'-C leads to form pi bond between 2' and 3' carbons of -1rA/G of the nick while the water molecule between 3'-OH of -1rA/G of the nick and D570 directs the water leaving group at 3'-C of -1rA/G and forms carbocation at the 3'-end (B and C). Then the nucleophilic attack from hydroxyl (OH) of 5'-P of +1G of the nick can form a phosphodiester bond between 5'-P of +1G and 3'-C of -1rA/G as shown in post-step 3 structures (D and E).

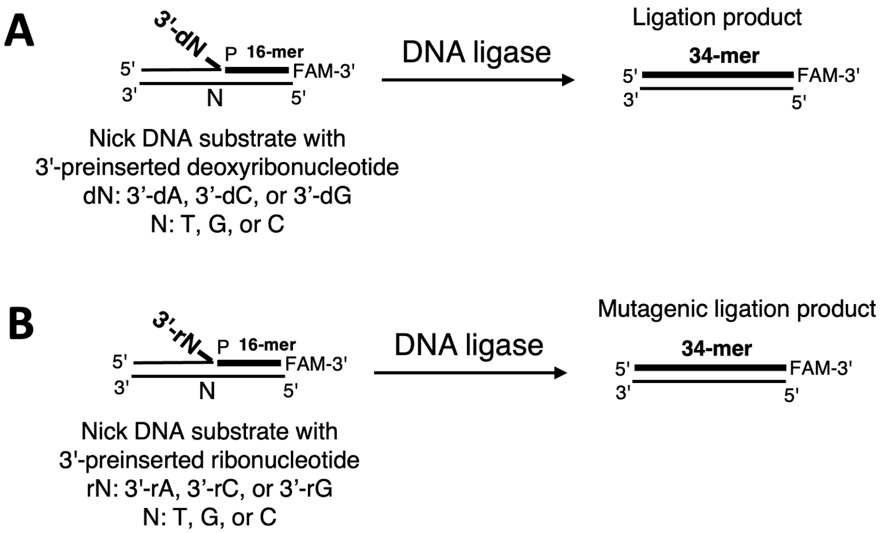

Supplementary Scheme 3. Illustration of DNA ligation assays used in this study. Ligation assays were used to investigate the nick sealing efficiency of DNA substrates containing preinserted Watson-Crick base-paired ends 3'-dA:T, 3'-dC:G, 3'-dG:C (**A**) and 3'-ribonucleotides 3'-rA:T, 3'-rC:G, 3'-rG:C (**B**).
